# Degradation of AMPK‐α1 sensitizes BRAF inhibitor‐resistant melanoma cells to arginine deprivation

**DOI:** 10.1002/1878-0261.12151

**Published:** 2017-11-16

**Authors:** Ying‐Ying Li, Chunjing Wu, Sumedh S. Shah, Shu‐Mei Chen, Medhi Wangpaichitr, Macus T. Kuo, Lynn G. Feun, Xiaoqing Han, Miguel Suarez, Jeffrey Prince, Niramol Savaraj

**Affiliations:** ^1^ Department of Medicine University of Miami Miller School of Medicine FL USA; ^2^ Sylvester Comprehensive Cancer Center University of Miami Miller School of Medicine FL USA; ^3^ Division of Hematology and Oncology Miami Veterans Affairs Healthcare System FL USA; ^4^ Dauer Electron Microscopy Lab Department of Biology University of Miami FL USA; ^5^ Department of Neurosurgery Taipei Medical University‐Wan Fang Hospital Taiwan; ^6^ Graduate Institute of Clinical Medical Sciences College of Medicine Chang Gung University Tao‐Yuan Taiwan; ^7^ Department of Surgery University of Miami Miller School of Medicine FL USA; ^8^ Department of Molecular Pathology University of Texas M. D. Anderson Cancer Center Houston TX USA; ^9^ Department of Laboratory Medicine Miami Veterans Affairs Healthcare System FL USA

**Keywords:** AMPK‐α1, autophagy, BRAF inhibitor‐resistant melanoma, E3 ubiquitin ligase, metabolic reprogramming, ubiquitin‐proteasome system

## Abstract

Melanomas harboring BRAF mutation (V600E) are known to recur frequently following treatment with BRAF inhibitors (BRAFi) despite a high initial response rate. Our previous study has uncovered that BRAFi‐resistant melanoma (BR) cells are vulnerable to arginine deprivation. It has been reported that naïve melanoma cells undergo autophagy and re‐express argininosuccinate synthetase 1 (ASS1) to enable them to synthesize arginine for survival when encountering arginine deprivation. Abolishing these two factors in BR cells confers sensitivity to arginine deprivation. In this report, we further demonstrated that downregulation of AMPK‐α1 in BR cells is a major factor contributing to impairment of autophagy as evidenced by decreased autophagosome formation. These BR cells also showed a metabolic shift from glucose to arginine dependence, which was supported by decreased expressions of GLUT1 (glucose transporter) and hexokinase II (HKII) coupled with less glucose uptake but high levels of arginine transporter CAT‐2 expression. Furthermore, silencing CAT‐2 expression also distinctly attenuated BR cell proliferation. Notably, when naïve melanoma cells became BR cells by long‐term exposure to BRAFi, a stepwise degradation of AMPK‐α1 was initiated *via* ubiquitin‐proteasome system (UPS). We discovered that a novel E3 ligase, RING finger 44 (RNF44), is responsible for promoting AMPK‐α1 degradation in BR cells. RNF44 expression in BR cells was upregulated by transcription factor CREB triggered by hyperactivation of ERK/AKT. High levels of RNF44 corresponding to low levels of AMPK‐α1 appeared in BR xenografts and melanoma tumor samples from BR and BRAFi/MEK inhibitor (MEKi)‐resistant (BMR) melanoma patients. Similar to BR cells, BMR cells were also sensitive to arginine deprivation. Our study provides a novel insight into the mechanism whereby BRAFi or BRAFi/MEKi resistance drives proteasomal degradation of AMPK‐α1 and consequently regulates autophagy and metabolic reprogramming in melanoma cells.

**Abbreviations**
ACC: acetyl‐CoA carboxylase
ADI‐PEG20: pegylated arginine deiminase
AMPK: AMP‐activated protein kinase
ASS1: argininosuccinate synthetase
BMR: BRAF inhibitor/MEK inhibitor resistant
BRAFi: BRAF inhibitor
BR: BRAF inhibitor resistant
CAT‐1/2: cationic amino acid transporter
ChIP: chromatin immunoprecipitation
CHX: cycloheximide
CREB: cAMP response element binding protein
GLUT1: glucose transporter 1
HCQ: hydroxychloroquine
HKII: hexokinase II
IHC: immunohistochemistry
LKB1: liver kinase B1
MAGE‐A3: melanoma‐associated antigen 3
MEKi: MEK inhibitor
mTOR: mechanistic target of rapamycin
RNF: RING finger
TEM: transmission electron microscopy
TRIM28: tripartite motif‐containing 28
UPS: ubiquitin‐proteasome system

## 1 Introduction

Approximately 50% of cutaneous melanomas have BRAF (V600E) mutation, and most of them respond well to BRAF inhibitor (BRAFi) treatment (vemurafenib or dabrafenib) (Chapman *et al*., 2011; Flaherty *et al*., 2010). Although the combination of BRAFi and MEK inhibitor (MEKi) can increase response rate and duration of response, most patients developed resistance to these treatments (Hauschild *et al*., 2012; Sosman *et al*., 2012). Numerous studies have found that several alternative pathways, such as activated COT, CRAF, IGF‐1R, PDGFRβ, and mutation of RAS, can bypass BRAF inhibition and subsequently trigger activation of downstream ERK/AKT (Nazarian *et al*., 2010; Poulikakos and Rosen, 2011; Villanueva *et al*., 2010). Nevertheless, these mechanisms occur in only 60% of progressing melanoma tumors. Additionally, resistance heterogeneity within melanoma tumors in patients presents a challenging clinical problem (Rizos *et al*., 2014; Shi *et al*., 2014). We have previously reported several unique changes related to bioenergetics which act in concert and make BRAFi‐resistant (BR) cells extremely vulnerable to arginine deprivation, regardless of whichever alternative signal pathways they utilize to evade the antitumor effect of BRAFi (Li *et al*., 2016). In this report, we have investigated the mechanisms leading to these changes.

Our previous studies have illustrated that arginine deprivation achieved by treatment with arginine deiminase (ADI‐PEG20) can suppress tumor growth in 70% melanomas due to their low or no expression of argininosuccinate synthetase 1 (ASS1), an essential enzyme needed to generate arginine from citrulline (Feun *et al*., 2008). Therefore, these melanoma cells lacking ASS1 expression must acquire exogenous arginine to support biosynthesis of polyamines and other amino acids for protein synthesis. Acquisition of exogenous arginine can be accomplished through cationic amino acid transporters CAT‐1 and CAT‐2 (Closs *et al*., 2004; Lu *et al*., 2013). In this communication, we have investigated alterations of these transporters in BR cells.

ADI‐PEG20, a mycoplasma enzyme converting exogenous arginine to citrulline and ammonia, has shown antitumor effects in many cancer types (Feun *et al*., 2008; Kelly *et al*., 2012; Miraki‐Moud *et al*., 2015; Qiu *et al*., 2014), yet cancer cells survive after this treatment due to ASS1 re‐expression and undergoing autophagy (Tsai *et al*., 2012; You *et al*., 2013). Previously, it has been shown that ASS1 re‐expression is due to upregulation of positive regulator c‐Myc after ADI‐PEG20 treatment (Tsai *et al*., 2012). Conversely, attenuated c‐Myc‐mediated ASS1 expression occurs in BR cells and therefore increases sensitivity to ADI‐PEG20 treatment (Li *et al*., 2016). To further enhance their ability to survive, naïve melanoma cells undergo autophagy through AMP‐activated protein kinase (AMPK) activation (Savaraj *et al*., 2010; Yang *et al*., 2011). Activated AMPK phosphorylates ULK directly or through mechanistic target of rapamycin (mTOR) inhibition and subsequently triggers autophagy (Jeyabalan *et al*., 2012; Savaraj *et al*., 2010; Yang *et al*., 2011).

LKB‐AMPK axis has been regarded as a master energy sensor. Loss of LKB1 has been shown to induce mouse embryonic fibroblast (MEF) apoptosis in response to nutrient stress due to inability to activate AMPK‐mediated autophagy (Shaw *et al*., 2004). In BRAF‐mutant melanoma cells, activated BRAF constitutively phosphorylates ERK and ribosomal s6 kinase (RSK) which phosphorylate LKB1 (Ser325 and Ser428) and in turn suppress its capability to activate AMPK in melanomas (Esteve‐Puig *et al*., 2009; Zheng *et al*., 2009). In contrast, another study revealed that BRAFi cannot restore LKB1‐AMPK activation and triggers LKB1‐AMPK‐independent autophagy (Ma *et al*., 2014). Therefore, whether BRAF‐ERK suppresses LKB1‐AMPK activation remains unclear in BR cells. Another means to regulate AMPK activity is through its degradation *via* ubiquitin‐proteasome system (UPS) (Zungu *et al*., 2011). Currently, it is known that AMPK is composed of α, β, and γ subunits. Recent data suggested that AMPK‐α1 (possessing Thr172 phosphorylation site) can be degraded by MAGE‐A3/6‐TRIM28, which leads to tumorigenesis (Pineda *et al*., 2015). Besides autophagy, AMPK is also a vital regulator of metabolism. Activated AMPK phosphorylates acetyl‐CoA carboxylase (ACC) that triggers fatty acid oxidation to generate energy and terminates fatty acid biosynthesis (Hardie and Pan, 2002). Stimulation of AMPK using agonists can enhance glucose uptake and glycolysis through upregulation of GLUT1 (glucose transporter) and hexokinase II (HK II) (Hardie, 2011; Hardie *et al*., 2012; Wu and Wei, 2012). Overall, AMPK is a major regulator of both metabolism and autophagy.

This study uncovered that BRAFi resistance downregulates AMPK activity through UPS‐mediated AMPK‐α1 degradation. We further identified RING finger 44 (RNF44), a novel E3 ligase responsible for AMPK‐α1 degradation. Downregulation of AMPK‐α1 switches glucose dependence toward arginine dependence *via* attenuated GLUT1 and significantly upregulated arginine transporter CAT‐2 expression. Under arginine starvation, ASS1‐negative BR cells are unable to efficiently utilize glucose, synthesize arginine, and undergo autophagy to survive. Hence, they are more sensitive to arginine deprivation than their parental counterparts.

## 2 Materials and methods

### 2.1 Cell lines and reagents

The BRAF‐mutant (V600E) melanoma cell lines were incubated with vemurafenib (Selleck Chemicals, Houston, TX, USA) over 30 weeks to generate BR cell lines. IC50 values of vemurafenib for parental and BR cells have been described in the previous study (Li *et al*., 2016). The parental cell lines (A375, A2058, UACC62, and SK‐MEL28) and skin fibroblast cell line BJ were purchased from American Type Culture Collection (ATCC, Manassas, VA, USA); MEL‐1220 and MEL‐DA were established in our laboratory. BRAFi/MEKi‐resistant (BMR) melanoma cell lines (A375BMR SK‐MEL28BMR, and A2058BMR) were created by incubating their parental cells with the combination of vemurafenib and IC50 values of trametinib (20 nm for A2058, 10 nm for A375, and 1 nm for SK‐MEL28). Except for SK‐MEL‐28 (cultured in DMEM; Thermo Fisher Scientific), all melanoma cell lines were cultured in MEM supplemented with 10% FBS (Atlanta Biologicals, Flowery Branch, GA, USA) and streptomycin/penicillin (Thermo Fisher Scientific, Waltham, MA, USA) in CO_2_ incubator.

For autophagy assay, 10 μm hydroxychloroquine (HCQ; Sigma‐Aldrich, St. Louis, MO, USA) was prepared in medium and used to treat parental and BR cells. To investigate the mechanism of proteasomal degradation, we treated the parental and BR cells with a proteasome inhibitor MG‐132 (Selleck Chemicals) or an inhibitor of protein synthesis [cycloheximide (CHX); Sigma‐Aldrich] to observe the turnover of AMPK‐α1. MEKi (trametinib/GSK1120212) and MK‐2206 were purchased from Selleck Chemicals, and SCH772984 was from APExBio (Houston, TX,USA). These compounds were utilized to inhibit phosphorylation of MEK, ERK, and AKT, respectively, to determine their role in RNF44 expression. The arginine‐free medium was generated by adding ADI‐PEG20 (100 ng·mL^−1^; Polaris Pharmaceuticals, San Diego, CA, USA) into complete MEM medium at 37 °C and incubating for 48 h.

### 2.2 Antibodies

For immunoblotting, antibody against ubiquitin (Ub), AMPK‐α1, p‐AMPK (Thr172), Akt, p‐Akt (Thr308), p‐Akt (Ser473), ERK1/2, LC3‐I/II, or p‐CREB (Ser133) was purchased from Cell Signaling Technology (Danvers, MA, USA). Anti‐MAGE‐A3, anti‐ASS1, anti‐p‐ERK, anti‐AMPK‐β, anti‐AMPK‐r, anti‐TRIM28, and anti‐CREB antibodies were separately, obtained from Abgent (San Diego, CA, USA), BD Biosciences (San Jose, CA, USA), Polaris, Sigma‐Aldrich, and GeneTex (Irvine, CA, USA). Anti‐RNF44 antibody was purchased from Abcam (Cambridge, MA, USA). All secondary antibodies conjugated with HRP were purchased from Promega (Madison, WI, USA). The immunoblots were developed using ultrasensitive enhanced chemiluminescent substrate and visualized by ChemiDoc MP System (Bio‐Rad, Hercules, CA, USA). The anti‐CREB antibody used for chromatin immunoprecipitation (ChIP) was purchased from Cell Signaling Technology.

For CAT‐1 and CAT‐2 detection, melanoma cells were incubated with primary antibody (CAT‐1, Novus, Littleton, CO, USA, 1 : 20; CAT‐2, Santa Cruz Biotechnology, Santa Cruz, CA, USA, 1 : 20) for 20 min and then incubated with second antibody conjugated with Alexa Fluor^®^ 555 (Thermo Fisher Scientific) for 20 min at room temperature. The samples were analyzed by FACS (BD Accuri™ C6).

### 2.3 Transfection of plasmids and RNA interference (RNAi)

The plasmid inserted with RNF44‐GFP or GFP gene was purchased from OriGene (Rockville, MD, USA) and then mixed with lipofectamine (Thermo Fisher Scientific) for transfection. Regarding knockdown experiment, individual nucleotides (siRNAs) targeting AMPK‐α1, RNF44, CAT‐2, and nontargeting (NT) control siRNAs were obtained from Dharmacon (Lafayette, CO, USA) and OriGene. These siRNAs were delivered into the cells using transfection reagent INTERFERrin (Polyplus, New York, NY, USA) according to the instruction of the manufacturer.

### 2.4 RNA analysis

Total RNA was extracted using Trizole reagent (Thermo Fisher Scientific) and converted into cDNA using iScript cDNA synthesis kit (Bio‐Rad). The cDNA was mixed with SYBR Green SuperMix reagent (Bio‐Rad) and gene‐specific PCR primers and analyzed by real‐time PCR analysis (CFX96; Bio‐Rad). The gene expression was normalized by GAPDH.

Primer sequences for AMPK‐α1 are 5′‐GGTCCATAGAGATTTGAAACCTG‐3′ (forward) and 5′‐GCCTGCATACAATCTTCCTG‐3′ (reverse); primer sequences for CAT‐1 are 5′‐CTTCATCACCGGCTGGAACT‐3′ (forward) and 5′‐GGGTCTGCCTATCAGCTCGT‐3′ (reverse); primer sequences for CAT‐2 are 5′‐TTCTCTCTGCGCCTTGTCAA‐3′ (forward) and 5′‐TCTAAACAGTAAGCCSTCCCGG‐3′ (reverse); primer sequences for RNF44 are 5′‐CCTACTTCCTCTCGATGCTG‐3′ (forward) and 5′‐CTGCTCTATGTCTGCTTTGG‐3′ (reverse); primer sequences for GAPDH are 5′‐CTCTCTGCTCCTCCTGTTC‐3′ (forward) and 5′‐GGTGTCTGAGCGATGTGG‐3′ (reverse).

### 2.5 Luciferase reporter assay

Before amplifying varying DNA fragments located in RNF44 promoter (−1785 to +120) using Bio‐Rad CFX PCR System and High‐Fidelity PCR SuperMix (Thermo Fisher Scientific), we designed seven primers including forward primers extended with *Kpn*I and a reverse primer extended with *Nhe*I for DNA amplification. The forward primer sequence for region −33 to +122 is 5′‐TATGCAGGTACCCGAGTGATTGGCTCTCAGGG‐3′; the forward primer sequence for region −118 to +122 is 5′‐TATGCAGGTACCGGTCCCTTTAAGTGCGAAGGT‐3′; the forward primer sequence for region −423 to +122 is 5′‐TATGCAGGTACCGTTGTTAGAGCCAGCATAACCA‐3′; the forward primer sequence for region −530 to +122 is 5′‐TATGCAGGTACCCGCCGTGTAGTATAAACAAGGAG‐3′; the forward primer sequence for region −1122 to +122 is 5′‐TATGCAGGTACCATTATTCGACCTGTTCCAGCTC‐3′; the forward primer sequence for region −1785 to +122 is 5′‐TATGCAGGTACCTGACCTTGCTCTTGTGTTGCT‐3′; the common reverse primer sequence at +122 is 5′‐ATTCGTGCTAGCTTGATTCACAACATTCGAAGCGG‐3′. The PCR products and pGL3‐based vector carrying luciferase (LUC) gene (Promega) were, respectively, digested with enzyme *Kpn*I/*Nhe*I. Afterward, DNA fragments and linear pGL3 plasmid were ligated together using T4 ligase (New England Biolab, Ipswich, MA, USA).

Regarding LUC activity assay, empty vector pGL3 and various RNF44 promoter constructs were delivered into melanoma cells using Lipofectamine (Thermo Fisher Scientific) for 6 h and then cultured in the presence of inhibitors for 24 h followed by detecting LUC activity using luciferase assay system kit (Promega).

### 2.6 Site‐directed mutagenesis and chromatin immunoprecipitation (ChIP)

For site‐directed mutagenesis, the sequences of mutagenic primers recognizing CREB binding sites are 5′‐GCGGTTAAATGTCTCTGTGATAGGAGCGCGAGCAGGGC‐3′ (first CREB binding site) and 5′‐GCAGCTCTTGGGGGTGATAGGATCTCCGGGAAGGTG‐3′ (second CREB binding site). The assay was carried out using the kit purchased from Agilent Technology (Santa Clara, CA, USA) per manufacturer's instruction.

The ChIP assay kit was purchased from Millipore (Burlington, MA, USA). The PCR primer sequences for first CREB binding site are 5′‐CGCCGTGTAGTATAAACAAGGAG‐3′ (forward) and 5′‐ACAGGGTGCCGCCTGAGATACT‐3′ (reverse); primer sequences for second CREB binding site are 5′‐AGGGAGGTCTCCGCGGGGAC‐3′ (forward) and 5′‐ACGAGCTAACGTCTGCCGGGC‐3′ (reverse).

### 2.7 Glucose uptake analysis

Melanoma cells were harvested and suspended in 100 μL PBS. Thereafter, cells were incubated with 2‐NBDG (Thermo Fisher Scientific, 100 μm) for 15 min at 37 °C and then analyzed by FACS BD Accuri™ C6.

### 2.8 Intracellular ATP detection

Both parental and BR melanoma cells were cultured in 24‐well plates and transfected with siRNA against CAT‐2 (OriGene) overnight. Afterward, these transfectants were transferred to 96‐well plates (1 × 10^4^/well) and incubated at 37 °C for 24 h. For ATP extraction, trichloroacetic acid (TCA, 1%) and 2 mm EDTA were added into transfectants and then were neutralized with 20 mm Tris/acetate (pH 7.8). Intracellular ATP concentration was detected using the ENLITEN^®^ ATP assay system bioluminescence detection kit (Promega) and normalized by protein content.

### 2.9 Detection of cell viability, apoptosis, and autophagy

5 × 10^3^ melanoma cells were incubated with various doses of ADI‐PEG20 (0–1000 ng·mL^−1^) for 72 h. The cell proliferation was analyzed by MTT (Sigma‐Aldrich). The IC50 values of ADI‐PEG20 in both parental and BR cells have been shown in our previous study (Li *et al*., 2016). Apoptosis was determined using caspase activity assay kit (ApoSat apoptosis detection kit; R&D System, Minneapolis, MN, USA). The apoptotic proportion was analyzed by FACS (Accuri™ C6; BD Biosciences). Lysotracker Red (Thermo Fisher Scientific) and Cyto‐ID (Enzo Life Sciences, Farmingdale, NY, USA) were applied to autophagosome staining.

### 2.10 Immunoprecipitation and proteomic analysis

Cell lysates were collected from parental and BR cells after incubated with or without ADI‐PEG20 (100 ng·mL^−1^) in the presence of MG‐132 (10 μm) for 4 h. Immunoprecipitation was completed by adding anti‐AMPK‐α1 antibody (Santa Cruz Biotechnology) and Gammabind plus Sepharose bead slurry (GE Healthcare, Life Science, Marlborough, MA, USA) into the cell lysates. Immunoprecipitates were subjected to SDS/PAGE and immunoblotting. For proteomic analysis, we sent the immunoprecipitates of A2058 and A2058BR cells to Applied Biomics, Inc. (San Francisco, CA, USA), to identify the putative proteins interacting with AMPK‐α1 using two‐dimensional difference gel electrophoresis (2D‐DIGE) and matrix‐assisted laser absorption ionization‐time of flight mass spectrometry (MALDI‐TOF‐MS). The data were analyzed using GPS explorer equipped with search engine MASCOT.

### 2.11 *In vivo*

The protocol of *in vivo* experiment has been reviewed and approved by the Institutional Animal Care and Use Committee (IACUC, #7715.63MR) at Miami VA Medical Center. 1 × 10^6^ cells were injected subcutaneously into female athymic nude‐Foxn1^nu^ mice (6‐8 weeks) purchased from Harlan Laboratories (Indianapolis, IN, USA). When the tumor volumes reached 100 mm^3^, the tumor‐bearing mice were randomly assigned to the control group or the experimental group. The experimental group received an intramuscular injection of ADI‐PEG20 (100 IU·kg^−1^), and the control group was treated with normal saline twice per week.

### 2.12 Immunohistochemical (IHC) staining

The tissue slides were dewaxed by xylene. Antigen retrieval was performed using citric acid (10 mm, pH 6.0). The tumor tissue slides were separately incubated with anti‐ASS1 (Polaris), anti‐RNF44 (Novus, 1 : 200), anti‐CAT‐1 (Novus, 1 : 50), anti‐CAT‐2 (Novus, 1 : 50), and anti‐AMPK‐α1 (Novus, 1 : 200) antibodies at 4 °C overnight. The slides then were stained with LSAB™2 Kits (DAKO, Carpinteria, CA, USA) and hematoxylin (DAKO) and visualized by a light microscope (Olympus, Center Valley, PA, USA). The levels of ASS1, RNF44, and AMPK‐α1 were randomly scored upon intensity scale ranging from 0 to 3+ and percentage of positive cells in tumor tissues. The outcome was based on scoring (*H*‐score) proposed by K.S. McCarty (McCarty *et al*., 1986). We were blinded to the allocation of pretreatment and post‐treatment groups in this experiment.

### 2.13 Transmission electron microscopy (TEM)

Briefly, cells were fixed in 2.5% glutaraldehyde at room temperature. The specimens were postfixed in 1% osmium tetroxide (OsO_4_) for 10 min, dehydrated using a graded ethanol series, en bloc stained with 2% uranyl acetate in 50% ethanol for 30 min, and embedded in Spurr's epoxy resin. Thereafter, ultrathin (< 90 nm) sections were cut using a Diatome 3‐mm diamond knife on the Leica EM UC6 ultramicrotome. The ultrathin sections were stained using lead citrate to be viewed under TEM on a Jeol 1400 EM at 80 kV.

### 2.14 Patients' samples and explant assay

Tumor tissues were obtained after obtaining informed consent approved by Institutional Review Board (#19990582) at the University of Miami Miller School of Medicine. With respect to explant assay, the tumor tissues obtained from patients were cut into small pieces (average diameter is < 0.2 cm) and then randomly seeded into transwells hanging on 24‐well plates (lower compartments) containing medium and drugs and incubated for 48–72 h.

### 2.15 Statistics

Statistical analysis was performed by Student's *t‐*test and assessed using GraphPad Prism (La Jolla, CA, USA). All data were shown as mean ± standard error of mean (SEM). Every result was completed by three independent experiments. *P*‐value < 0.05 was regarded as significant difference.

## 3 Results

### 3.1 BRAFi resistance switches metabolism toward arginine addiction in melanoma cells

We have generated five BR cell lines and three BMR cell lines from parental cell lines (A375, A2058, MEL‐1220, SK‐MEL‐28, and UACC‐62) harboring BRAF (V600E) mutation by long‐term exposure to IC50 values of vemurafenib or in combination with trametinib as stated in Table S1.

**Figure 1:**
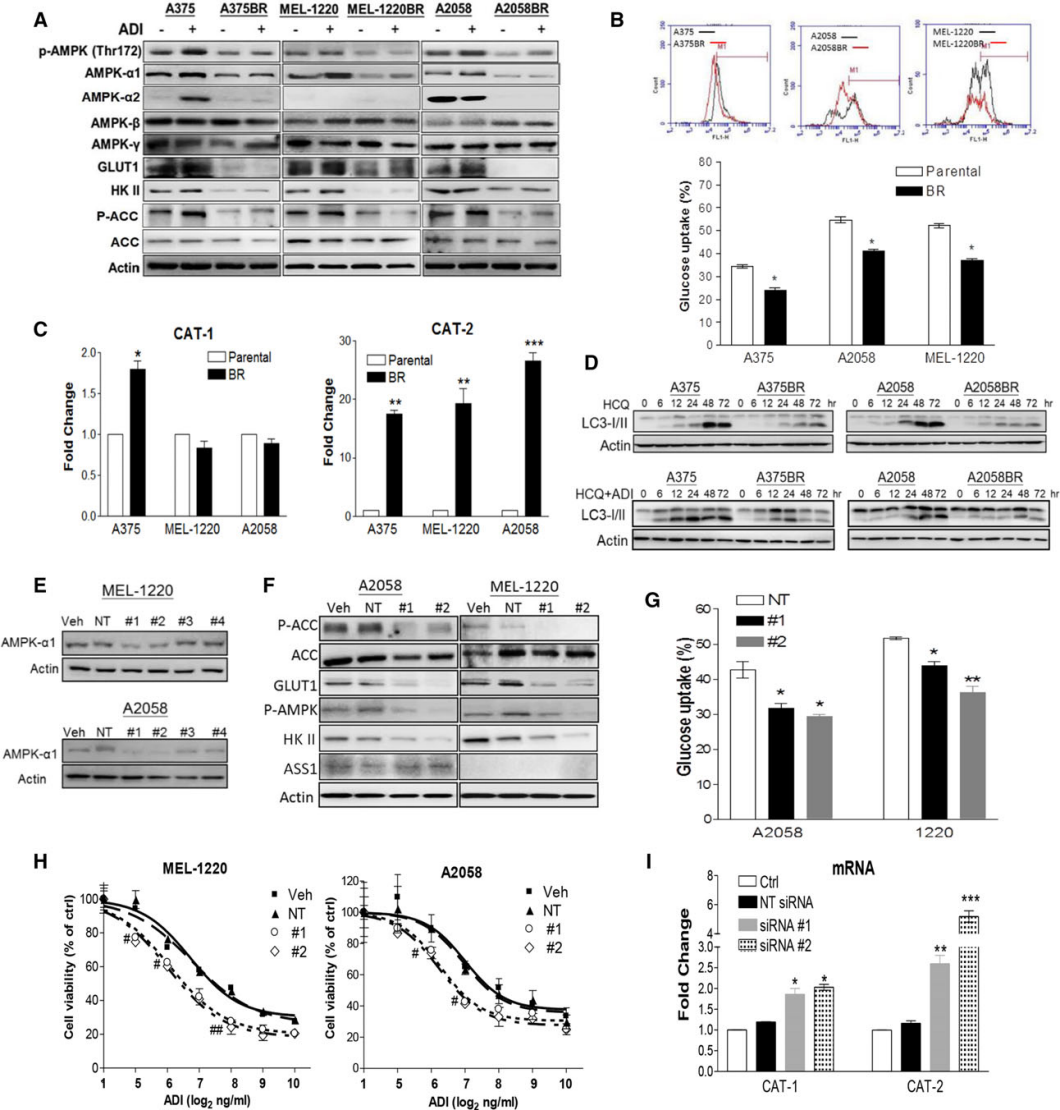
BRAFi resistance results in metabolic reprogramming toward arginine addiction and circumvention of autophagy due to downregulation of AMPK‐α1. Parental and BR cells were incubated with ADI‐PEG20 (100 ng·mL^−1^) for 72 h (A) or HCQ alone with or without for 0–72 h (D). Their cell lysates were subjected to immunoblotting. (B) Glucose uptake activity in parental and BR cells was analyzed by 2‐NBDG uptake using flow cytometry (FACS). (C) RNA levels of CAT‐1 and CAT‐2 were detected by qRT‐PCR. On the other hand, MEL‐1220 and A2058 cells were transfected with individual siRNAs (50 nm) against AMPK‐α1, nontargeting (NT) siRNA, or transfection reagent alone (vehicle, Veh). These transfectants were analyzed using immunoblotting (E,F), glucose uptake (G), MTT assay (^#^ *P* < 0.05 and ^##^ *P* < 0.01) (H) and qRT‐PCR (I). Data are represented as mean ± SEM (*n* = 3, *P < 0.05, **P < 0.01, and ***P < 0.005). Fig. 1A reproduced from our previous study (Li *et al*., 2016).

A previous study has shown that BR cells switch dependence on glycolysis to mitochondrial oxidative phosphorylation (Baenke *et al*., 2016). To examine whether low activity of glycolysis is also seen in our BR cells, we determined glucose uptake and key enzymes in the glycolytic pathway. The results substantiated that GLUT1 and hexokinase II (HK II) were downregulated in BR cells (Fig. 1A), corresponding to less glucose uptake assayed by 2‐NBDG compared to parental cells (Fig. 1B). Our previous study has reported that BR cells are extremely auxotrophic for arginine due to downregulated c‐Myc‐mediated ASS1 re‐expression (Li *et al*., 2016). Therefore, we hypothesized that these BR cells failing to re‐express ASS1 to generate arginine must increase the ability to acquire exogenous arginine through upregulation of arginine transporters CAT‐1 and CAT‐2 to survive. To test this hypothesis, we first examined whether ASS1 appearance affects expressions of CAT‐1 and CAT‐2 in A375 and A2058 cells (ASS1‐negative cells). The plasmid carrying ASS1 described in the previous publication (Long *et al*., 2013) was delivered to these two cell lines. The result showed that ASS1 overexpression significantly downregulated CAT‐2 rather than CAT‐1 in these melanoma cells (Fig. S1A,B). We next observed their expressions in parental cells and BR cells. As expected, BR clearly exhibited higher CAT‐2 expression compared to their parental cells (Fig. 1C and Fig. S2). To verify whether arginine acquisition mediated by CAT‐2 is critical for ATP synthesis, CAT‐2 expression in A2058BR and A375BR cells was silenced using individual siRNAs, and intracellular ATP concentration has been analyzed. Consistent with the previous study reported by Baenke *et al*. (2016), the basal levels of ATP in BR cells were much higher than those in parental counterparts due to the fact that BR cells utilized mitochondrial oxidative phosphorylation instead of glycolysis to generate ATP (Fig. S1C). Ablation of CAT‐2 remarkably reduced ATP synthesis as well as cell viability in BR cells (Fig. S1C,D). Collectively, our findings suggested that BR cells are dependent on arginine more than glycolysis as their energy source.

### 3.2 BRAFi resistance‐downregulated AMPK‐α1 expression renders melanoma cells sensitive to arginine depletion

Our data showed that BR cells engaged in the acquisition of exogenous arginine instead of utilizing glucose. Both BR and BMR cells underwent apoptosis while encountering arginine depletion achieved by ADI‐PEG20 (100 ng·mL^−1^) treatment as shown by a drastic increase in caspase activity (Table S1). Moreover, ASS1 expression in some cell lines (e.g., A2058, SK‐MEL‐28, and UACC‐62) can be induced upon ADI‐PEG20 treatment and hence contributes to resistance to arginine depletion. An impaired ability to re‐express ASS1 in their BR/BMR cell lines increased sensitivity to ADI‐PEG20 treatment (Table S1) (Li *et al*., 2016). However, other cell lines, such as A375, MEL‐1220, and their BR/BMR cell lines do not have inducible ASS1, yet their BR/BMR cells were more sensitive to ADI‐PEG20 treatment. Thus, CAT‐2‐mediated arginine acquisition is critically important and may be regulated by other molecules in these ASS1‐negative BR/BMR cells. Besides dependency on arginine acquisition, attenuated autophagy in these BR/BMR cells could be the contributory factor of hypersensitivity to ADI‐PEG 20 treatment.

Our previous data have shown that naïve melanoma cells undergo autophagy to survive arginine deprivation (Savaraj *et al*., 2010), whereas BR cells lose this ability (Li *et al*., 2016). To support these results, both parental and BR cells were treated with HCQ (10 μm) to inhibit autolysosome formation by increasing pH in lysosomes and result in autophagosome accumulation presented by an increase in LC3‐I/II conversion. Our data depicted that LC3‐ll expression increased over time, and LC3‐II accumulation was faster in parental cells rather than in BR cells following treatment with HCQ. Although the combination of HCQ and ADI‐PEG20 enhanced LC3‐I/II expression, LC3‐II expression remained lower in BR cells and was diminished after 48 h due to undergoing apoptosis (Fig. 1D). Furthermore, ADI‐PEG20 treatment promotes p‐AMPK and AMPK‐α1 expression in parental cells, but lower levels of p‐AMPK and AMPK‐α1 were seen in BR cells (Fig. 1A). Thus, our results suggested that lower levels of AMPK‐α1 disable BR cells to survive by undergoing autophagy under arginine deprivation, and hence, they succumb to apoptosis.

### 3.3 AMPK‐α1 is crucial to governing metabolic reprogramming and autophagy in BR cells

Our previous study reported that AMPK activation in BR cells is p‐LKB independent (Li *et al*., 2016). Low levels of p‐AMPK (Thr172) correlated with low levels of AMPK‐α1 (possessing Thr172), but not with AMPK‐α2, β, and γ subunits, resulting in downregulated p‐ACC expression (Fig. 1A). As AMPK has been reported to regulate autophagy, glucose uptake, and glycolysis (Hardie and Pan, 2002; Hardie *et al*., 2012), we next examined whether downregulation of AMPK‐α1 can abort glycolysis and ADI‐PEG20‐induced autophagy in parental melanoma cells as seen in BR cells. The results demonstrated that knockdown of AMPK‐α1 using #1 or #2 siRNA (Fig. 1E) in parental cells yielded greater sensitivity to ADI‐PEG20 treatment when compared to nontargeting (NT) siRNA or vehicle (Fig. 1H). These transfectants also had less ability to undergo autophagy as evidenced by decreased LC3‐II expression and autophagosome formation upon arginine starvation (Fig. S3B,C). In regard to metabolic regulation, silencing AMPK‐α1 attenuated glucose uptake and disturbed fatty acid metabolism through downregulation of GLUT1 and p‐ACC, but did not affect ASS1 expression (Fig. 1F,G). Interestingly, arginine transporter CAT‐2 expression is also greatly increased by silencing AMPK‐α1 in parental cells, while only slightly enhanced CAT‐1 expression is detected (Fig. 1I and Fig. S3D). In contrast, BR cells transfected with AMPK‐α1 (PRKAA1)‐GFP‐overexpressed plasmid possessed higher GLUT1 expression compared to vehicle (GFP groups) (Fig. S4A). Moreover, *in vitro* study also confirmed that PRKAA1‐GFP overexpression restored autophagy in BR cells and hence rendered BR cells resistant to arginine depletion (Li *et al*., 2016). This evidence was further confirmed by the xenograft model demonstrating that (PRKAA1)‐GFP overexpression subverted ADI‐PEG20‐induced apoptosis in A2058BR by enhancing autophagosome formation (Fig. S4C,D). Furthermore, PRKAA1 overexpression strikingly attenuated RNA and protein levels of CAT‐2 in A2058BR and MEL‐1220BR cells and xenografts (Figs S4 and S5B). Although AMPK‐α1 overexpression slightly suppressed CAT‐1 in A2058BR xenografts, the basal levels of CAT‐1 were low so that there was no significant reduction seen in RNA levels. Overall, our data suggested that downregulated AMPK‐α1 expression in BR cells not only abrogates the autophagy but also switches metabolism to arginine addiction.

### 3.4 Downregulated AMPK‐α1 expression in BR melanoma cells is modulated by UPS

**Figure 2:**
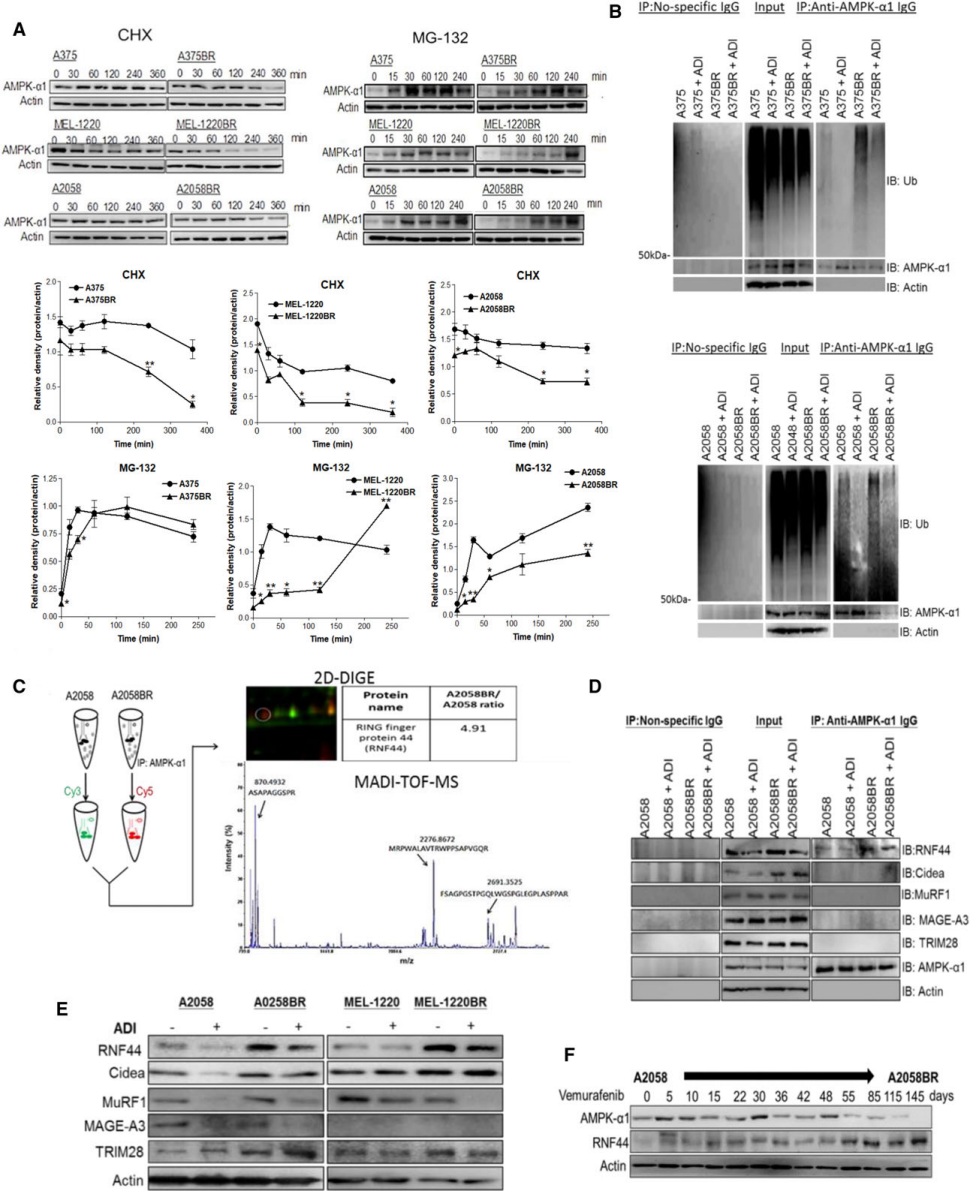
The E3 ligase RNF44 is implicated in UPS of AMPK‐α1 degradation in BR cells. (A) Parental cell and BR cells were incubated with MG‐132 (10 μm) and CHX (80 μg·mL^−1^), respectively. Cell lysates were collected at different time intervals, and subsequently, AMPK‐α1 was assayed by immunoblotting. The levels of AMPK‐α1 were quantitated by ImageJ and presented as curves. (B) Parental and BR cells were incubated with or without ADI‐PEG20 in the presence of MG‐132 (10 μm) for 4 h. Ubiquitin (Ub) and AMPK‐α1 were separately detected by immunoblotting following immunoprecipitation of AMPK‐α1. (C) The precipitated proteins from A2058 cells and A2058BR cells were, respectively, labeled with Cy3 and Cy5 and then were subjected to 2D gel. Thereafter, the Cy5‐positive spot was identified as RNF44 (Q7L0R7) by MALDI‐TOF MS based on UniProtKB/Swiss‐Prot database. (D) The levels of different E3 ligases (RNF44, Cidea, MAGE‐A3, and MuRF1) in precipitated proteins (D) or total cell lysates (E) were detected by immunoblotting. (F) A2058 cells were constantly treated with vemurafenib (5 μm), and its cell lysates were collected at different time points. The levels of AMPK‐α1, RNF44, or actin were detected by immunoblotting.

Low protein levels of AMPK‐α1 have been found in BR cells (Fig. 1A) and did not correlate with RNA levels (Fig. S3A). Thus, we speculated that more active AMPK‐α1 protein degradation occurs in BR cells. We treated parental and BR cells with proteasome inhibitor MG‐132 or a protein synthesis inhibitor CHX followed by immunoblotting of AMPK‐α1. As shown in Fig. 2A, compared with parental cells, BR cells displayed a delayed accumulation of AMPK‐α1 expression following treatment with MG‐132 but the faster degradation of AMPK‐α1 after CHX treatment. Furthermore, more ubiquitins were co‐immunoprecipitated with AMPK‐α1 in BR cells relative to parental cells, even in the presence of ADI‐PEG20 (Fig. 2B). Taken together, our results verified that BRAFi resistance enhances activation of AMPK‐α1 degradation through ubiquitin‐proteasome system (UPS).

### 3.5 Proteomic analysis discovers that a novel E3 ligase, RNF44, accounts for UPS of AMPK‐α1 degradation in BR cells

We then sought for *ad hoc* proteins implicated in UPS using immunoprecipitation of AMPK‐α1 followed by proteomic analyses. Notably, our proteomic analyses identified a novel protein, RNF44, which was 4.9‐fold higher in A2058BR immunoprecipitates relative to A2058 immunoprecipitates (Fig. 2C). Even though RNF44 has been categorized in the RING finger family, its biological functions have not been identified yet. Hence, we searched for putative proteins sharing similar peptide sequences with RNF44 in UniProKB/Swiss‐Prot database and then found E3 ligases RNF38 and praja‐1 (< 30% similarity) (Fig. S6A). Additionally, higher RNF44 expression seen in BR cell lysates can be co‐immunoprecipitated with AMPK‐α1 compared to parental cells even in the presence of ADI‐PEG20 (Fig. 2D,E). The time‐course experiment showed that RNF44 levels increased stepwise and inversely correlated with AMPK‐α1 levels when A2058 cells were exposed to BRAFi and gradually became BR cells (Fig. 2F). Currently, several possible ubiquitin ligases of AMPK‐α1 including Cidea, MuRF1, and MAGE‐A3/A6‐TRIM28 have been reported to ubiquitinate AMPK‐α or β in muscle cells, adipose tissue, cervical cancer, lung cancer, and colon cancer cells (Pineda *et al*., 2015; Zungu *et al*., 2011). However, none of these candidate proteins correlated with AMPK‐α1 expression and cannot be co‐immunoprecipitated with AMPK‐α1 in BR cells (Fig. 2D,E).

**Figure 3:**
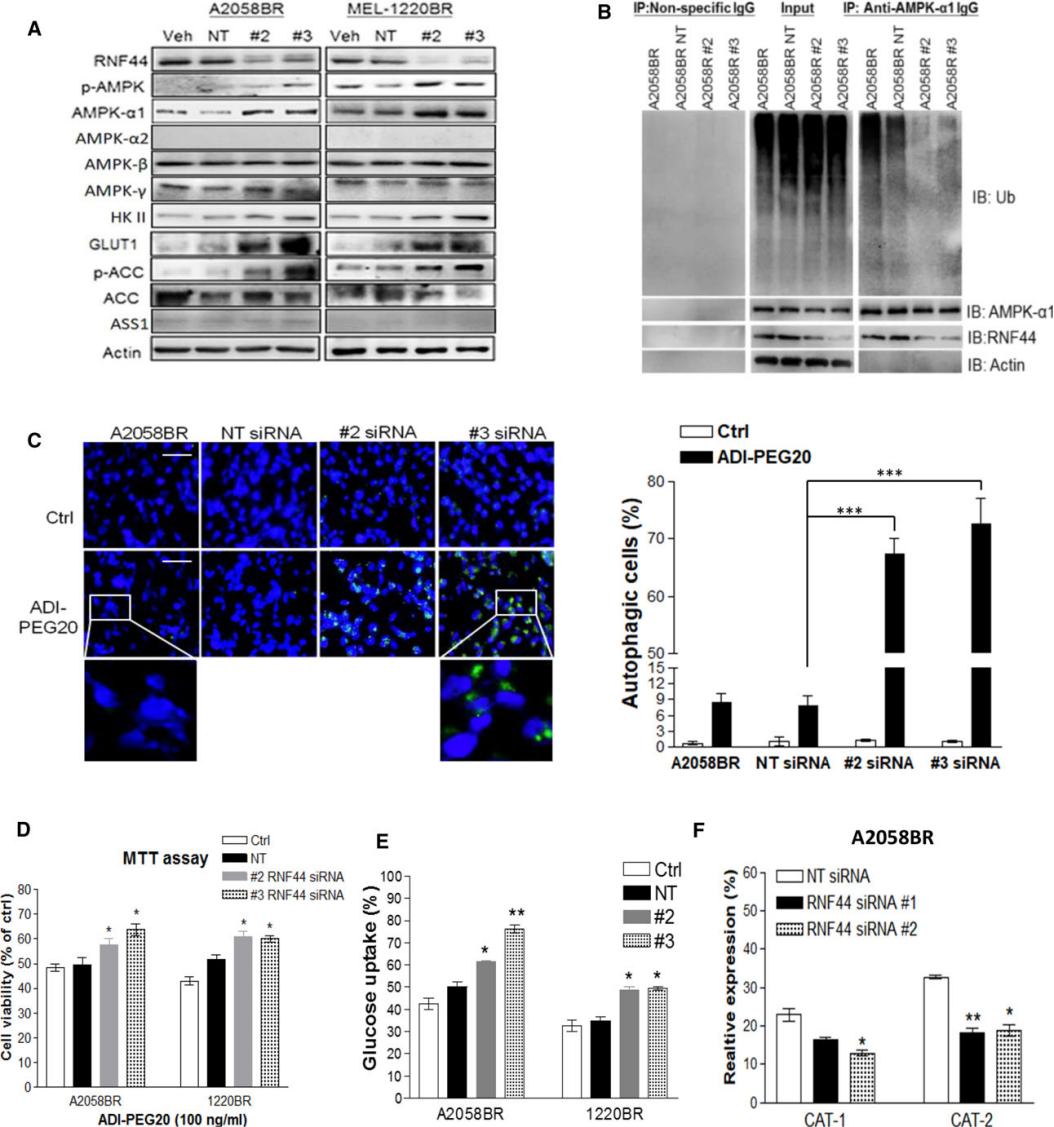
Silencing RNF44 in BR cells abrogates AMPK‐α1 degradation, switches metabolism to glucose addiction, and restores autophagosome formation upon arginine deprivation. BR cells were transfected with individual siRNAs against RNF44, nontargeting (NT) siRNA (50 nm), or transfection reagent alone (Veh, as a control group). Total cell lysates of these transfectants were subjected to immunoblotting (A) and immunoprecipitation of AMPK‐α1 (B). (C) A2058BR cells were transfected with RNF44 siRNAs and treated with ADI‐PEG20. Their autophagosomes and nuclei were separately stained with Cyto‐ID (green) and Hoechst 33342 (blue) and then visualized using fluorescent microscope (scale bar = 50 μm). Autophagy‐positive cells were quantitated and presented as a bar graph. (D) Cell viability of these transfectants was analyzed by MTT after treatment with ADI‐PEG20 (100 ng·mL^−1^) or completed medium for 48–72 h. (E) Glucose uptake was analyzed by 2‐NBDG uptake with FACS. (F) CAT‐1 and CAT‐2 expressions were assessed by FACS, and data were shown in a bar graph (*n* = 3, *P < 0.05, **P < 0.01, and ***P < 0.005).

To investigate whether RNF44 is involved in ubiquitination of AMPK‐α1, RNF44 expression in A2058BR cells was silenced using siRNAs. The result illustrated that silencing RNF44 expression enhanced AMPK‐α1 by abolishing its ubiquitination, yet it cannot be applied to the expression of α2, β, or γ subunit (Fig. 3A,B). As a result, these transfectants regained resistance to ADI‐PEG20 treatment by triggering autophagosome formation (Fig. 3C,D). For metabolic alteration, silencing RNF44 resulted in enhanced glucose uptake and fatty acid oxidation as evidenced by upregulation of AMPK‐mediated GLUT1 and HKII expressions and ACC phosphorylation, attenuated arginine transporter CAT‐2 expression, but did not affect ASS1 expression (Fig. 3A,E,F). Conversely, A2058 cells transfected with RNF44‐GFP‐overexpressed plasmid exhibited robust ubiquitination of AMPK‐α1 and consequently resulted in sensitization to arginine deprivation (Fig. S6B–D). Collectively, our data illustrated that RNF44 participates in AMPK‐α1 degradation in BR melanoma cells, resulting in downregulation of autophagy and glucose uptake but upregulation of CAT‐2. These biochemical alterations make melanoma cells hypersensitive to arginine deprivation.

**Figure 4:**
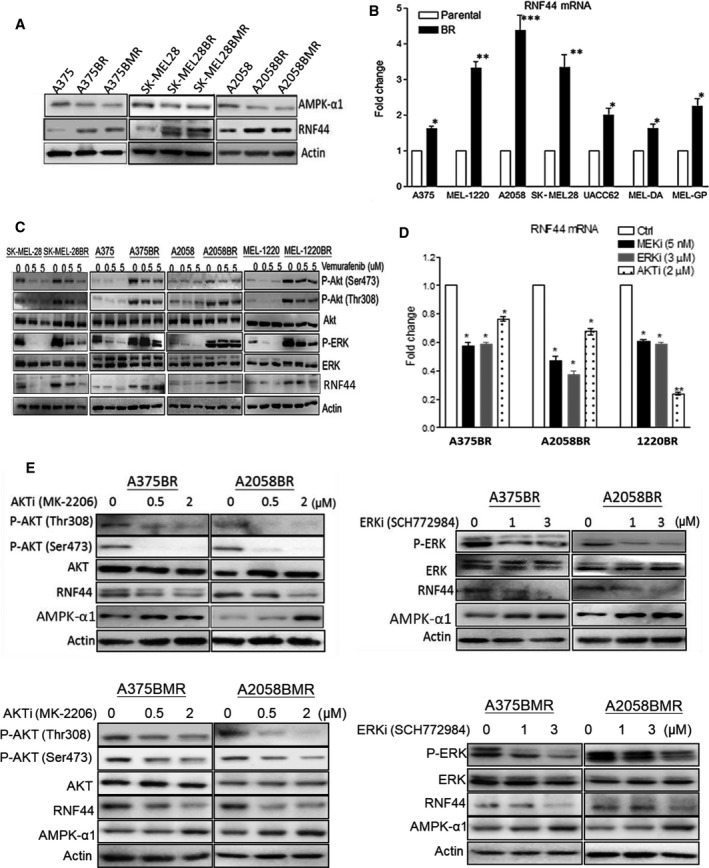
Upregulated RNF44 expression is related to hyperactivation of ERK and AKT in BR cells. (A) Immunoblotting demonstrated that BR and BMR cells possessed downregulated AMPK‐α1 and upregulated RNF44. (B) RNA levels of RNF44 in parental and BR cell lines were determined by qRT‐PCR and normalized by GAPDH. (C) Higher protein levels of p‐AKT, p‐ERK, and RNF44 appeared in BR cells even in the presence of BRAFi (vemurafenib). (D) BR cell lines were treated with MEKi (trametinib, 5 nm), ERKi (SCH772984, 3 μm), or AKTi (MK‐2206, 2 μm) for 24 h, and their RNF44 levels were determined by qRT‐PCR. Data shown in a bar graph were represented as mean ± SEM (*n* = 3, *P < 0.05, **P < 0.01, and ***P < 0.005). (E) Protein levels of ERK, AKT, AMPK‐α1, and RNF44 in BR and BMR cells were determined by immunoblotting following treatment with AKTi or ERKi.

Currently, there is no published literature on how RNF44 is upregulated in BR cells. As higher levels of RNF44 seen in BR cells may be due to increased transcription or translation, we determined RNA levels of RNF44 by qRT‐PCR. Our results showed that higher RNF44 RNA levels contributed to higher RNF44 protein levels (Fig. 4A,B). As the majority of BR cells expressed high levels of phosphorylated ERK/AKT (Fig. 4C) (Welsh *et al*., 2016), we hypothesized that ERK/AKT hyperactivation in BR cells may upregulate RNF44 expression. To test this hypothesis, we treated BR cells with ERK inhibitor (ERKi, SCH772984), MEKi (trametinib), and AKT inhibitor (AKTi, MK‐2206) and determined RNF44 levels. The results revealed that AKT or ERK inhibition attenuated RNF44 expression in A375BR and A2058BR cells (Fig. 4D,E). This correlation can also be seen in A375BMR and A2058BMR cells. Our findings suggested that ERK/AKT hyperactivation may contribute to elevated RNF44 expression, leading to ubiquitination of AMPK‐α1.

### 3.6 BRAFi resistance‐induced RNF44 transcription in melanoma cells is mainly triggered by transcription factor CREB

**Figure 5:**
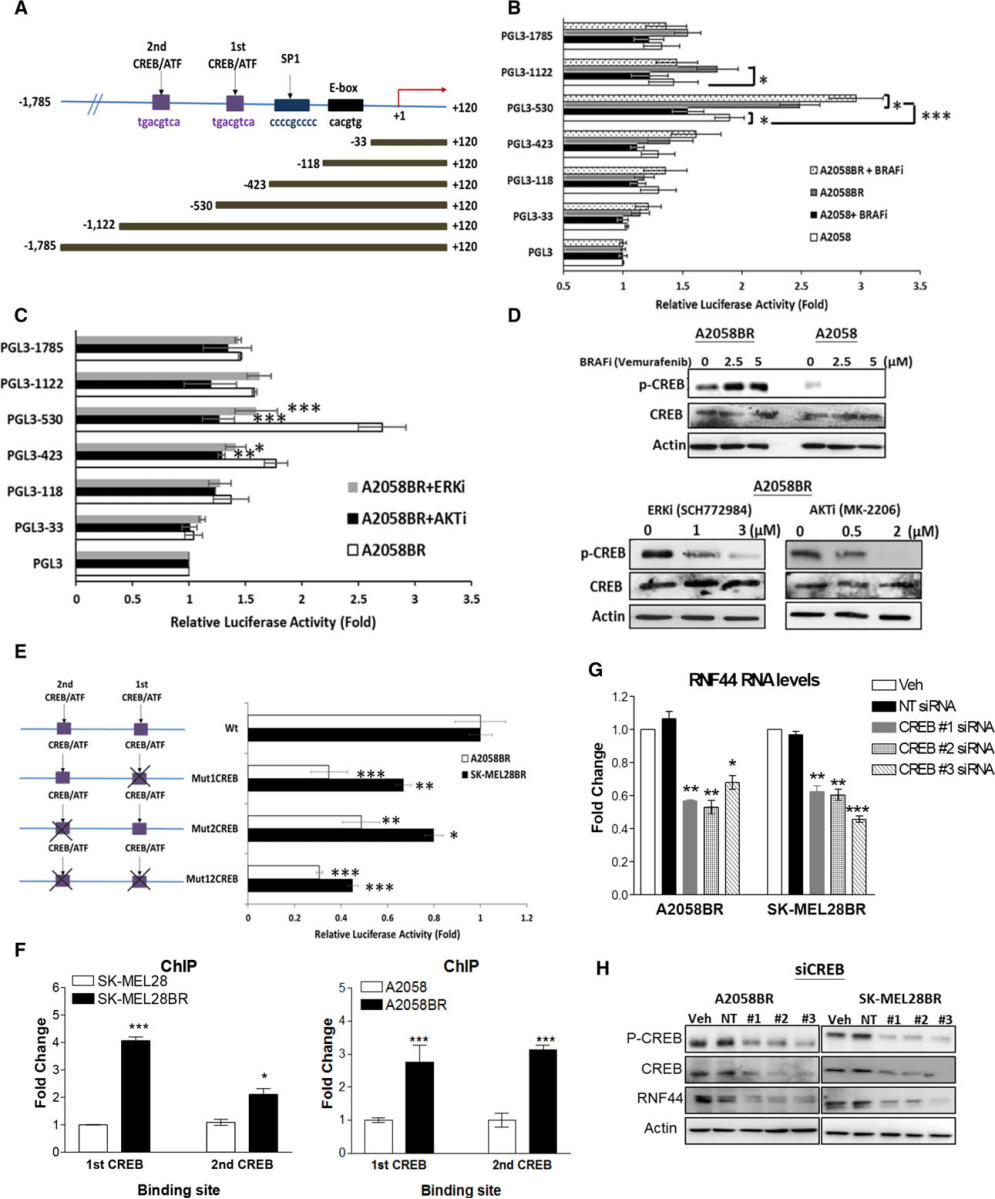
ERK/AKT‐activated CREB triggers transcription *via* two cis‐regulatory elements of RNF44. (A) Delineation of RNF44 promoter region (−1785 to +120) and its deletions. (B,C) A2058 and A2058BR cells transfected with pGL3 luciferase vectors carrying various fragments of RNF44 promoter regions were treated with or without BRAFi (5 μμ), ERKi (3 μμ), or AKTi (2 μμ). The luciferase activity was shown in bar graphs (*n* = 3, *P < 0.05, **P < 0.01, and ***P < 0.005). (D) Immunoblotting displayed the levels of p‐CREB (Ser133) and CREB in A2058BR cells treated with BRAFi, ERKi, and AKTi. (E) Four different mutant constructs established from pGL3‐1122 vector were created by site‐directed mutagenesis and then were, respectively, delivered into A2058BR and SK‐MEL28BR cells. The bar graph represented luciferase activity normalized by wild‐type (Wt). (F) Chromatin immunoprecipitation (ChIP) assay. The cell lysates extracted from A2058/A2058BR and SK‐MEL28/SK‐MEL28BR cells were incubated with antibody against CREB and with nonspecific IgG, and their binding sites in RNF44 promoter region were analyzed by real‐time PCR. The data have been normalized by nonspecific IgG and their parental counterparts. (G,H) CREB was silenced using siRNAs, RNF44 RNA levels were determined by qRT‐PCR, and protein levels were analyzed by immunoblotting (*n* = 3, *P < 0.05, **P < 0.01, and ***P < 0.005).

As we have demonstrated that resistance to BRAFi initiates RNF44 transcription, we next examined the underlying transcriptional regulation of RNF44. Firstly, we predicted that RNF44 promoter region contains E‐box, GC‐box (Sp1 binding site), and two CREB binding sites (Fig. 5A) based on decipherment of DNA elements (DECODE) database. Secondly, we constructed pGL3 LUC reporter vectors with various lengths of RNF44 promoter region and in turn detected transcriptional activity by LUC reporter assay. The result revealed that the LUC activity of pGL3‐530 containing first CREB binding site was highest among RNF44 promoter constructs in A2058BR cells incubated with BRAFi (Fig. 5B). Moreover, it has been known that CREB can be activated by RAF/ERK/RSK and AKT through phosphorylation at Ser133 (Chang *et al*., 2003; Du and Montminy, 1998). Hence, we speculated that BRAFi resistance‐induced hyperactivation of ERK/AKT elicits more p‐CREB (Ser133) to initiate RNF44 transcription. Thirdly, we determined p‐CREB (Ser133) expression in A2058 and A2058BR cells treated with or without BRAFi. As expected, the addition of BRAFi resulted in enhanced p‐CREB expression in A2058BR cells but downregulated p‐CREB in A2058 cells (Fig. 5D). To further test whether inhibition of ERK or AKT deters CREB from triggering RNF44 transcription, A2058BR transfected with various constructs was treated with AKTi or ERKi. The LUC activity of pGL3‐530 and the levels of p‐CREB were dramatically decreased by AKT or ERK antagonist (Fig. 5C,D).

To examine whether CREB binding sites mainly govern RNF44 transcription activity in BR cells, site‐directed mutagenesis of these binding sites was carried out and their pGL3 constructs are shown in Fig. 5E. The LUC activity disclosed that mutation (Mut) occurring either first or second CREB binding site resulted in lower transcription activity than wild‐type (Wt). Mutating two CREB binding sites further reduced more LUC activity compared to single mutant CREB binding site. To verify whether these vital binding sites are highly recognized by CREB in BR cells, antibody against CREB, or nonspecific IgG (as a negative control), was applied to ChIP followed by real‐time PCR assay. The result showed that more CREB binding sites can be co‐immunoprecipitated with CREB in BR cells (Fig. 5F). Moreover, knockdown of CREB resulted in lower RNA and protein levels of RNF44 in BR cells (Fig. 5G,H). Altogether, the results suggested that RNF44 expression is primarily controlled by CREB binding at its binding sites.

### 3.7 *In vivo* and *ex vivo* models confirm inverse correlation between RNF44 and AMPK‐α1 in BR and BRAFi/MEKi‐resistant (BMR) melanomas

**Figure 6:**
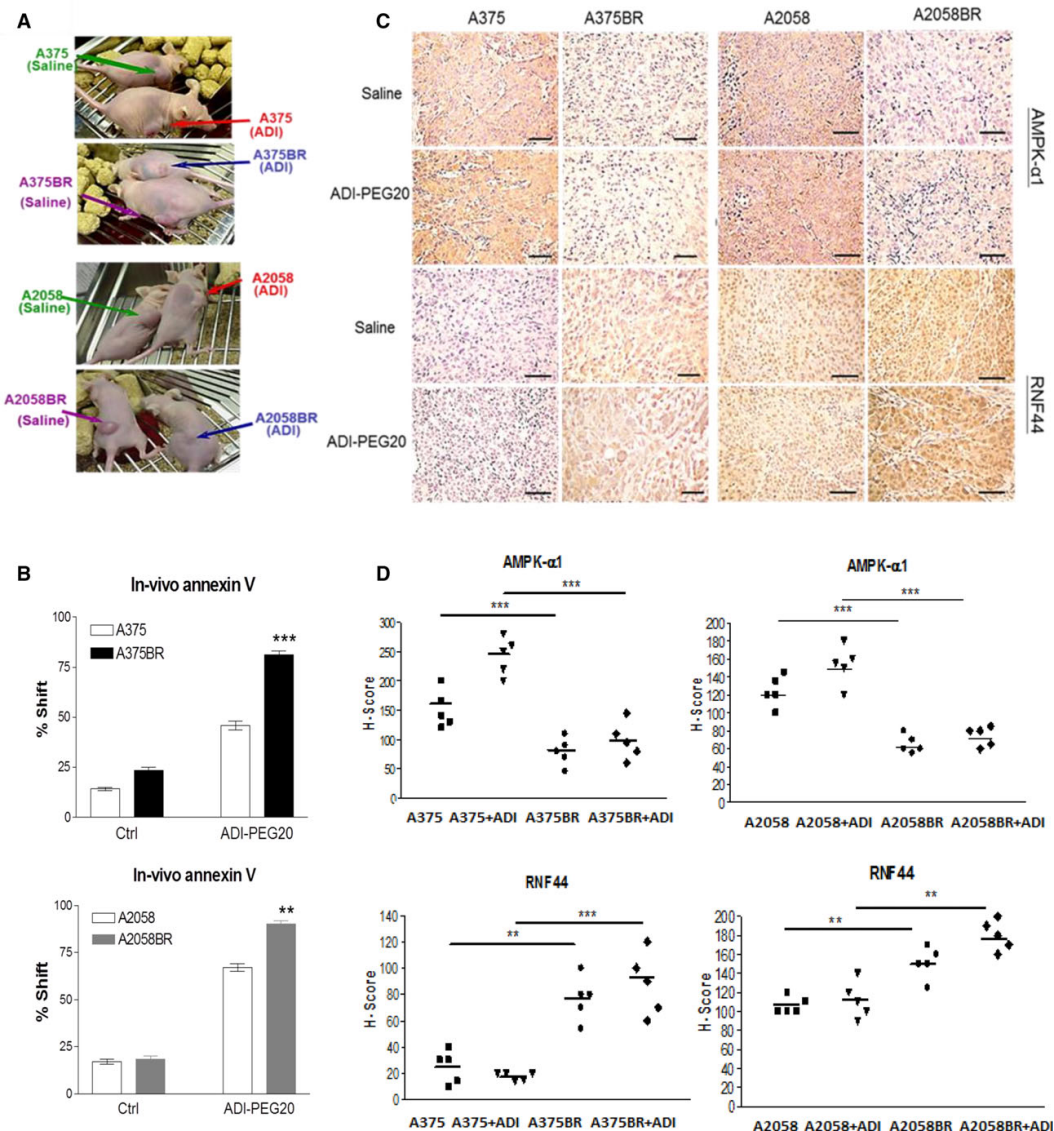
Upregulated RNF44 and downregulated AMPK‐α1 sensitize BR xenograft tumors to ADI‐PEG20 treatment. (A,B) Corresponding to our previous study (Li *et al*., 2016), ADI‐PEG20 significantly reduced BR tumor sizes by inducing cell apoptosis. Female nude mice were inoculated subcutaneously with 1 × 10^6^ melanoma cells. When tumor volume reached 100 mm^3^, tumor‐bearing mice received treatment of ADI‐PEG20 (100 IU·kg^−1^) twice per week. On day 32, single‐cell suspensions were prepared from tumor tissues and then were labeled with Annexin V and then analyzed by FACS. (C) (D) AMPK‐α1 and RNF44 expressions were detected by IHC staining, and the data were presented as *H*‐scores (scale bar = 100 μm) (*n* = 5, **P < 0.01, and ***P < 0.005).

Our previous data showed that treatment with ADI‐PEG20 is able to abort growth of BR xenograft tumors but only slow the growth of parental xenograft tumors (Li *et al*., 2016) (Fig. 6A). Furthermore, increased apoptosis in A2058BR and A375BR cells treated with ADI‐PEG20 was verified by *in vivo* Annexin V analysis (Fig. 6B). Consistent with *in vitro* data, *H*‐scores representing AMPK‐α1 levels were 1.5‐ to 2.5‐fold lower in BR xenograft tumors, while RNF44 levels were 1.4‐ to 4‐fold higher compared to parental xenograft tumors (Fig. 6C,D).

**Figure 7:**
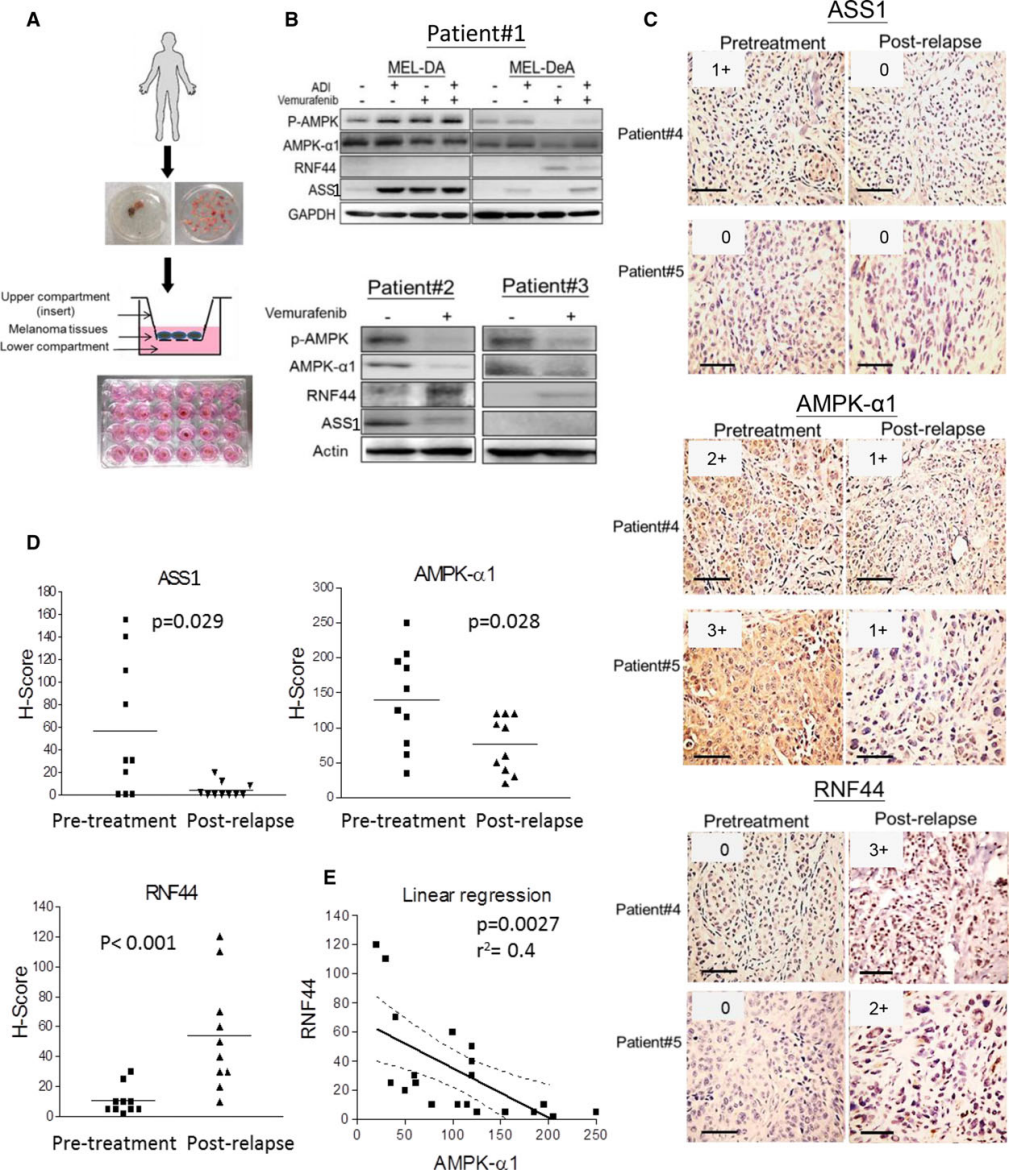
Attenuated ASS1 and AMPK‐α1 but increased RNF44 levels were seen in tumors from BR/BMR patients. (A) The explant assay was shown as schematic workflow, which has been described in Section 2.10. (B) Tumor explants from melanoma patients (patients #2 and #3) who relapsed following treatment with vemurafenib were placed in the inserts and exposed to drugs (in lower compartments) for 48–72 h. The cell lysates were subjected to immunoblot analysis. MEL‐DA and MEL‐DeA cells were primary cultures isolated from a melanoma patient #1 and represent pretreatment and BRAFi resistance (postrelapse), respectively. (C) ASS1, AMPK‐α1, and RNF44 expressions in paraffin‐embedded tumor tissues of patient #4 (resistant to BRAFi) and patient #5 (resistant to BRAFi/MEKi) were determined by IHC staining (scale bar = 100 μm). The intensities were scored and shown in upper right corner of each image. (D) The dot plots represented *H*‐scores of ASS1, AMPK‐α1, and RNF44 in tumor samples from melanoma patients with pretreatment (*n* = 10) and BRAFi or BRAFi/MEKi resistance (*n* = 10). (E) The correlation between RNF44 and AMPK‐α1 levels was assessed by a linear regression.

Attenuated AMPK‐α1 and increased RNF44 levels were also found in melanoma tumors from patients who failed BRAFi or BRAFi/MEKi treatment. Primary cultures including MEL‐DA (pretreatment) and MEL‐DeA (postrelapse) were incubated with vemurafenib and/or ADI‐PEG20. The levels of p‐AMPK/AMPK‐α1 and ASS1 were upregulated in MEL‐DA, but downregulated in MEL‐DeA following vemurafenib treatment. Treatment with ADI‐PEG20 enhanced phosphorylation of AMPK, but levels of RNF44 were not detectable in MEL‐DA (Fig. 7B). In contrast, RNF44 expression was detected after vemurafenib treatment in MEL‐DeA, corresponding to decreased levels of p‐AMPK/AMPK‐α1. We suggested that BR primary cultures may lose this correlation over time in the absence of vemurafenib, but it could be evoked by the addition of vemurafenib. The explants from BR patient #2 and patient #3 also displayed similar results as MEL‐DeA (Fig. 7A,B). Immunohistochemistry (IHC) results also confirmed that BRAFi resistance and BRAFi/MEKi resistance attenuated the levels of AMPK‐α1 in patient #4 (BR) and patient #5 (BMR) (Fig. 7C). Prior to BRAFi treatment, low expression of ASS1 can be seen in tumor tissue from patient #4, but not from patient #5. However, ASS1 expression completely disappeared in relapsed tumor #4. Furthermore, expression of ASS1, AMPK‐α1, or RNF44 represented as *H*‐score confirmed that BR/BMR tumor samples had 2‐7‐fold lower ASS1 and AMPK‐α1 than the average levels but fivefold higher RNF44 compared to naïve melanoma samples (Fig. 7D). RNF44 expression can be seen in both nuclei and cytoplasm (Fig. S7) and also inversely correlated with AMPK‐α1 (Fig. 7E). These correlations also appeared in BMR cell lines (A2058BMR and SK‐MEL28BMR) established *in vitro* (Fig. 4A). These BMR cells were also sensitive to ADI‐PEG20 treatment (Table S1).

## 4 Discussion

The combination of BRAFi and MEKi has been approved by the FDA as first‐line treatment for BRAF‐mutant melanoma patients. Although this combination can prolong progression‐free survival, patients eventually relapse due to MEK2 mutation‐ and BRAF amplification‐induced ERK reactivation (Sosman *et al*., 2012; Villanueva *et al*., 2013). Other combination treatments, such as BRAFi in combination with HCQ, HSP90 inhibitor, CDK4/6 inhibitor, and mTOR inhibitor, have been shown to improve antitumor effects *in vitro* and *in vivo* (Acquaviva *et al*., 2014; Fedorenko *et al*., 2015; Paraiso *et al*., 2012), yet their clinical efficacy remains unproven.

We have demonstrated that most BR cells are extremely vulnerable to arginine deprivation (Li *et al*., 2016). Our results further uncovered that BR cells have attenuated levels of c‐Myc and AMPK‐α1, which are crucial to governing ASS1 re‐expression, metabolic reprogramming, and autophagy. Additionally, c‐Myc and AMPK‐α1 have been shown to promote glucose uptake and glycolysis *via* upregulation of GLUT1 and HK II (Hardie, 2011; Miller *et al*., 2012). Indeed, our results substantiated that BR cells have lower glucose uptake as well as decreased HK II and GLUT1 expressions (Fig. 1A,B). To compensate for this, BR cells lacking ASS1 consume amino acids such as exogenous arginine by increasing arginine transporter CAT‐2 to generate ATP *via* oxidative phosphorylation (OXPHOS) (Baenke *et al*., 2016; Hernandez‐Davies *et al*., 2015) (Fig. 1C, Figs S1 and S2C,D), and hence, the cells are vulnerable to arginine‐depleting agents. In addition to arginine, other amino acids, such as glutamine, have been appreciated to be another energy source to sustain OXPHOS in mitochondria of melanoma cells when they develop resistance to BRAFi. Hence, glutamine transporter ASCT2 (anti‐neutral amino acid transporter) has been proposed as a potential therapeutic target in melanoma cells (Wang *et al*., 2014). Although *in vitro* study has shown that ASCT2 antagonist BenSer can suppress melanoma cell proliferation *via* cell cycle arrest and mTOR inhibition, this compound is not yet available in the clinic and its toxicity is unknown.

With respect to autophagic mechanism, our previous study elucidated that besides AMPK, a key autophagic protein involved in membrane elongation of autophagosome formation, Atg5, was also downregulated in BR cells (Li *et al*., 2016). Downregulation of Atg5 seen in BR cells may not sufficiently accomplish autophagy flux upon arginine depletion even in the presence of AMPK. However, our result demonstrated that restoring or overexpressing AMPK‐α1 in BR cells can regain the ability to undergo autophagy as evidenced by increased autophagosome formation under arginine starvation (Li *et al*., 2016) (Fig. 3C and Fig. S4D). Moreover, a previous study discovered that mammalian cells can utilize AMPK to trigger Atg5‐independent macroautophagy for mitochondrial clearance (Ma *et al*., 2015). In MEF, ULK1/2 which can be activated by AMPK triggers Atg5‐independent pathway through Rab9‐mediated lipidation instead of LC3‐II‐mediated lipidation (Nishida *et al*., 2009). It is possible that AMPK overexpression in BR cells initiates ULK1/2 and subsequently activates Atg5‐independent autophagy. Our previous study also disclosed that BR cells express equal levels of p‐ULK as parental cells; however, unlike parental cells, arginine depletion fails to enhance p‐ULK expression in BR cells due to the lack of AMPK‐α1 (Li *et al*., 2016). Taken together, our data suggest that low Atg5 expression in BR cells can contribute to its inability to undergo autophagy; nevertheless, the primary contributor to defective autophagy in BR cells is AMPK‐α1, a known master regulator of autophagy.

It has been reported that BRAF‐mediated ERK/RSK activation negatively regulates LKB through phosphorylation at Ser428 and subsequently inhibits activation and phosphorylation of AMPK at Thr172 (Zheng *et al*., 2009). However, LKB‐AMPK activation in melanoma cells cannot be restored by adding BRAFi (Ma *et al*., 2014). Therefore, AMPK‐α1 stability may govern AMPK activation. Indeed, a recent study has shown that ubiquitination on AMPK‐α1 can suppress LKB‐phosphorylated AMPK activation, and deubiquitinase USP10 can abort this process (Deng *et al*., 2016). Our previous data showed that elevated p‐LKB expression appears in BR cells, yet p‐AMPK and AMPK‐α1 attenuations are still present in BR cells following ADI‐PEG20 treatment (Li *et al*., 2016). Thus, our results illustrated that UPS rather than BRAF‐ERK regulates AMPK activity in BR cells.

Our results demonstrated that BR cells are much less capable of undergoing autophagy due to downregulation of AMPK‐α1 through active UPS (Fig. 2A,B). With regard to ubiquitination of AMPK in other tissue, MuRF1 has been experimentally demonstrated to add atypical ubiquitin chains on AMPK (Zungu *et al*., 2011). In brown adipose and heart tissues, Cidea regulates AMPK activity via interaction with AMPK‐β (Zungu *et al*., 2011). A recent study reported that a cancer‐specific ubiquitin ligase, MAGEA3/6‐TRIM28, participates in AMPK‐α1 degradation leading to inhibition of autophagy in HeLa, HEK‐293, and U2OS cells (Pineda *et al*., 2015). Nevertheless, none of these ubiquitin ligases can be detected to interact with AMPK‐α1 in BR melanoma cells (Fig. 2D). It is likely that ubiquitin ligases governing degradation of AMPK‐α1 are both tissue and tumor type specific. Our proteomic analyses have identified a novel protein, RNF44, which is a RING finger protein of C3H4 family. The actual structure needs to be confirmed by X‐ray crystallography. Notably, RNF44 can be detected in BR or BMR melanoma tissues rather than naive samples, and its expression also inversely correlates with AMPK‐α1 in tissue samples. Interestingly, BR cells, which have less ability to undergo autophagy due to attenuation of AMPK‐α1 secondary to higher levels of RNF44, also have high levels of p‐ERK/AKT. Blockade of AKT/ERK activation further confirmed that elevated RNF44 levels in BR cells are related to hyperactivation of AKT/ERK. The downstream transcription factor CREB, which has been reported to be activated/phosphorylated by AKT/ERK at Ser133, can interact with RNF44 promoter region and subsequently trigger RNF44 transcription (Fig. 5A–H).

## 5 Conclusion

**Figure 8:**
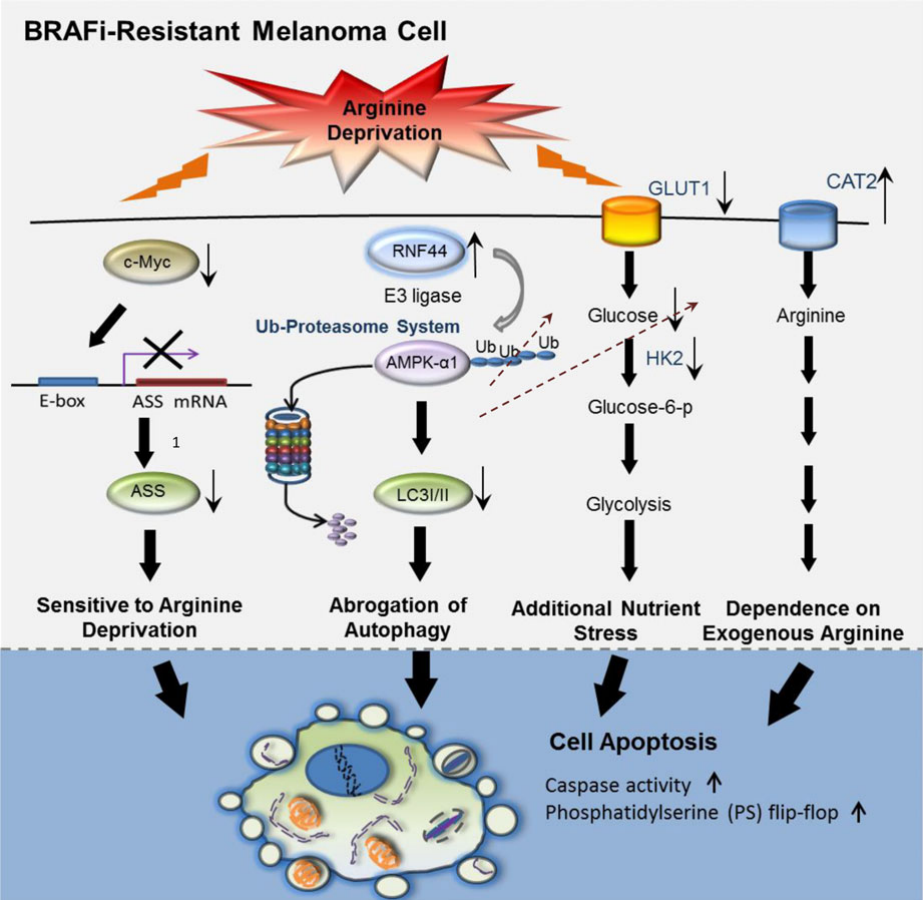
The schematic diagram illustrates several mechanisms leading to sensitivity to arginine deprivation/ADI‐PEG20 treatment in BR cells. BRAFi resistance downregulates c‐Myc‐mediated ASS1 transcription; hence, BR cells are arginine auxotrophic. Moreover, upregulated E3 ligase RNF44 also promotes AMPK‐α1 degradation, which abrogates autophagic flux, impairs glucose uptake and glycolysis, and enhances expression of arginine transporter 2 (CAT‐2). These biological alterations switch metabolism toward exogenous arginine addiction and consequently give rise to an increase in vulnerability to arginine deprivation in BR cells.

Resistance to BRAFi or BRAFi/MEKi enhances RNF44‐mediated AMPK‐α1 degradation. Downregulation of AMPK‐α1 disables autophagy and results in less glucose uptake but increases arginine transporter CAT‐2 expression (graphical abstract, Fig. 8). In addition, c‐Myc attenuation is known to attenuate glucose addiction as evidenced by a decrease in GLUT1 and HK II expressions (Long *et al*., 2013; Miller *et al*., 2012), and abrogate arginine synthesis due to downregulated ASS1 expression. In this circumstance, exogenous arginine becomes the energy source for BR cells. Thus, depleting arginine coupled with an inability to trigger autophagy and ASS1 expression drives BR cells to undergo apoptosis. Therefore, ADI‐PEG20 treatment could be a good candidate for salvage therapy in BR/BMR melanomas.

## Author contributions

YL and CW performed proliferation, apoptotic analyses, IP, immunoblotting, and creating BR cell lines. YL, SC, SS, and JP completed autophagosome and IHC staining. YL, SC, CW, and MW contributed to the results of animal study. XH and MK contributed to PGL3 plasmid and LUC activity assay. MS dictated H&E staining. YL, LF, NS, and SS participated in explant assay and composed and proofed the manuscript.

## Supporting information


**Fig. S1.** Arginine transporter CAT‐2 is needed for ASS1‐negative melanoma cells to obtain exogenous arginine for energy source.
**Fig. S2.** BRAFi resistant (BR) melanoma cells acquire more exogenous arginine by expressing higher levels of arginine transporter CAT‐2 compared to their parental counterparts.
**Fig. S3.** Silencing AMPK‐α1 expression results in abrogation of autophagy and significant upregulation CAT‐2 expression and slightly enhances CAT‐1 expression in parental cells.
**Fig. S4.** Overexpressing AMPK‐α1 (PRKAA1)‐GFP in BR cells restores the ability to undergo autophagy and switches acquisition of arginine to glucose.
**Fig. S5.** Mouse xenograft models demonstrate that overexpression of AMPK‐α1 (PRKAA1) in A2058BR cells drastically reduces CAT‐2 expression, corresponding to Fig. S4.
**Fig. S6**. Overexpressing RNF44 in A2058 cells renders A2058 cells more sensitive to arginine deprivation due to enhanced ubiquitin‐dependent AMPK‐α1 degradation.
**Fig. S7.** RNF44 expression appears in tumor tissues from patients who failed BRAFi (patient #4) or BRAFi/MEKi (patient #5) treatment, corresponding to Fig. 7C.
**Table S1.** Sensitivity of parental and BR melanoma cell lines to ADI‐PEG20.
