# Supplementary material for: Degradation of AMPK‐α1 sensitizes BRAF inhibitor‐resistant melanoma cells to arginine deprivation
Source: Mol Oncol. 2017 Nov 16;11(12):1806–25. doi: 10.1002/1878-0261.12151 (PMC5709618; doi:10.1002/1878-0261.12151)
Supplement: Supplementary file 1 — Fig. S1. Arginine transporter CAT‐2 is needed for ASS1‐negative melanoma cells to obtain exogenous arginine for energy source. Fig. S2. BRAFi resistant (BR) melanoma cells acquire more exogenous arginine by expressing higher levels of arginine transporter CAT‐2 compared to their parental counterparts. Fig. S3. Silencing AMPK‐α1 expression results in abrogation of autophagy and significant upregulation CAT‐2 expression and slightly enhances CAT‐1 expression in parental cells. Fig. S4. Overexpressing AMPK‐α1 (PRKAA1)‐GFP in BR cells restores the ability to undergo autophagy and switches acquisition of arginine to glucose. Fig. S5. Mouse xenograft models demonstrate that overexpression of AMPK‐α1 (PRKAA1) in A2058BR cells drastically reduces CAT‐2 expression, corresponding to Fig. S4. Fig. S6. Overexpressing RNF44 in A2058 cells renders A2058 cells more sensitive to arginine deprivation due to enhanced ubiquitin‐dependent AMPK‐α1 degradation. Fig. S7. RNF44 expression appears in tumor tissues from patients who failed BRAFi (patient #4) or BRAFi/MEKi (patient #5) treatment, corresponding to Fig. 7C. Table S1. Sensitivity of parental and BR melanoma cell lines to ADI‐PEG20. [file MOL2-11-1806-s001.pdf]

Table S1. Sensitivity of parental and BR melanoma cell lines to ADI-PEG20

Cell line	BRAF (V600E)	ASS1 level [a]	IC50 of ADI-PEG20[b]	Caspases activity (%)[c]	IC50 of BRAFi[b]	IC50 of MEKi
A375	Mut	(-) → (-)	181±10.5 ng/ml	27.2±3.1%	0.5±0.2 μM	9.8±1.1 nM
A375BR	Mut	(-) → (-)	68±8.2 ng/ml	50.3±7.8%	10.2±0.5 μM	5.6±0.8 nM
A375BMR	Mut	(-) → (-)	59± 6.6 ng/ml	55.1±6.3%	> 10 μM	17.1±2.1 nM
A2058	Mut	(-) → (+)	425±18.5 ng/ml	3.2±0.5%	5.1±0.4 μM	19±2.1 nM
A2058BR	Mut	(-) → (-)	125±7.8 ng/ml	27.9±4.8%	25±1.3 μM	15.3± 1.4 nM
A2058BMR	Mut	(-) → (-)	105±4.5 ng/ml	33.4±8.2%	> 25 μM	33.1±1.8 nM
MEL-1220	Mut	(-) → (-)	215±12 ng/ml	2.6±1.1%	4.2±0.5 μM	7.8±1.2 nM
MEL-1220BR	Mut	(-) → (-)	62.5±3.3 ng/ml	26.5±3.3%	15.2±0.8 μM	5.5±0.6 nM
SK-MEL-28	Mut	(-) → (+)(+)	222±4.3 ng/ml	9.8±2.3%	2.5±0.8 μM	1.2±0.3 nM
SK-MEL-28BR	Mut	(-) → (+)	103±8.4 ng/ml	20.5±3.7%	10±1.3 μM	2.0±0.5 nM
SK-MEL-28BMR	Mut	(-) → (-)	78.9±6.3 ng/ml	31.4±2.2%	> 10 μM	5.2±1.1nM
UACC-62	Mut	(-) → (+)	168±7.3 ng/ml	2.9±1.5%	7.8±0.6 μM	2.5±0.3 nM
UACC-62BR	Mut	(-) → (-)	92±5.5 ng/ml	14.9±5.5%	17.2±2.1 μM	1.9±0.5 nM
BJ	Skin fibroblast	(+)(+)→(+)(+)	ND	ND	ND	ND

[a] The inducible ASS expression has been identified by qRT-PCR and immunoblotting following ADI-PEG20 treatment (100 ng/ml) for 48 hr.

[b] IC50 values are based on cell viability curves, corresponding to previous study (Li, et al., 2016).

[c] The percentages of caspase activity were quantified by FACS following ADI-PEG20 treatment (100 ng/ml) for 72 hr. The proportions of treatment groups are normalized by those of untreated groups.

ND = Not detected, Mut = Mutant, BRAFi = vemurafenib, MEKi = trametinib

Suppl. Fig. 1

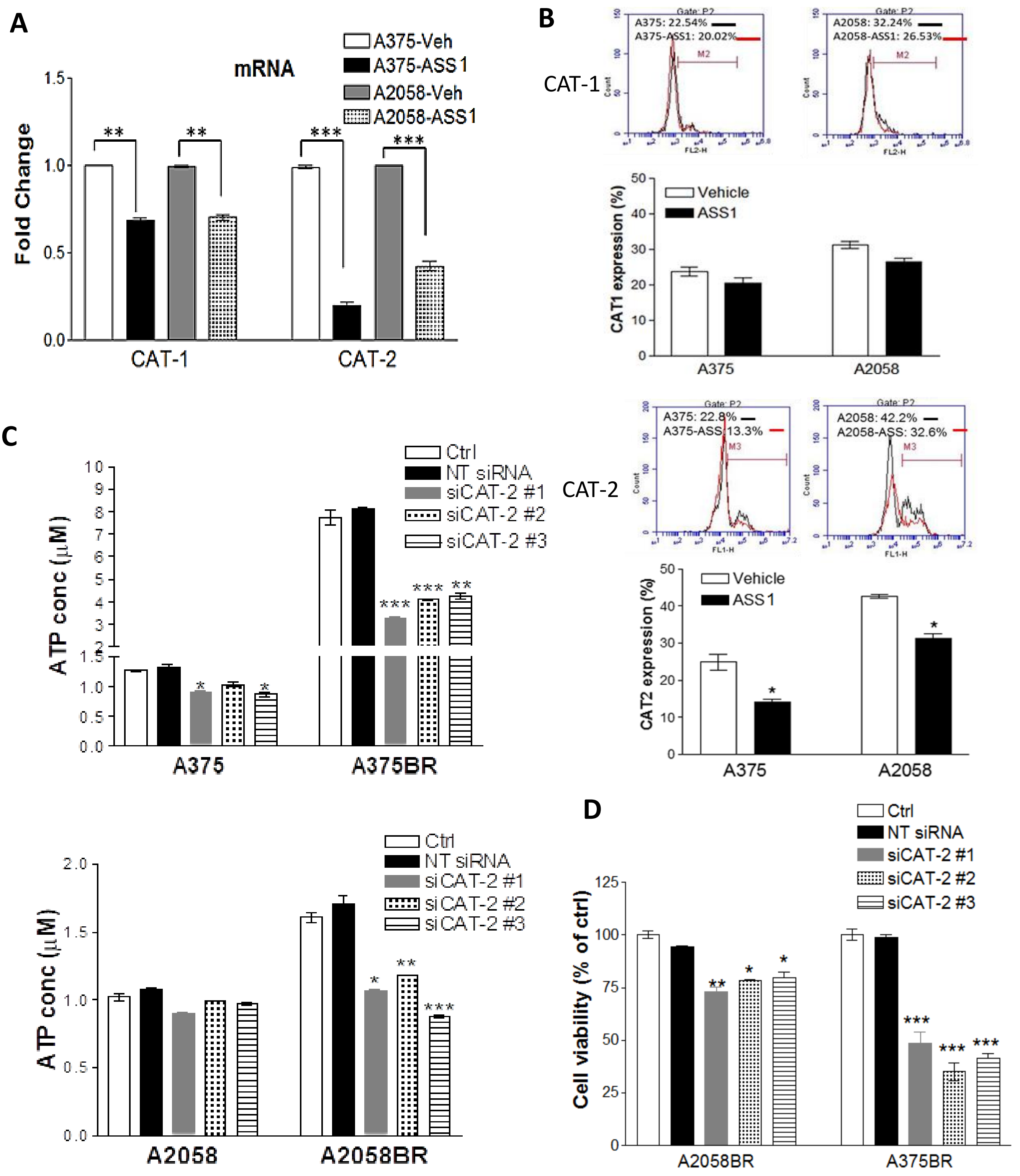

Suppl. Fig. 1. Arginine transporter CAT-2 is needed for ASS1-negative melanoma cells to obtain exogenous arginine for energy source. A375 and A2058 cells were transfected with the plasmid encoding ASS1 and vehicle plasmid, respectively. (A) CAT-1 and CAT-2 RNA levels were analyzed by qRT-PCR. (B) CAT-2 expression was detected by FACS and quantitated upon the percentage of right shift (M2 or M3 gate). (C) Knockdown of CAT-2 using 50 nM of siRNA (siCAT-2 #1, siCAT-2 #2, or siCAT-2 #3) was carried out in parental and BR cells for 48 hr and then their intracellular ATP concentration was detected. Intracellular ATP concentration was normalized by protein content. Non-targeting (NT) siRNA was used as a negative control. (D) The viability of BR cells was determined by MTT assay (*$p < 0.05$, **$p < 0.01$, and ***$p < 0.005$).

Suppl. Fig. 2

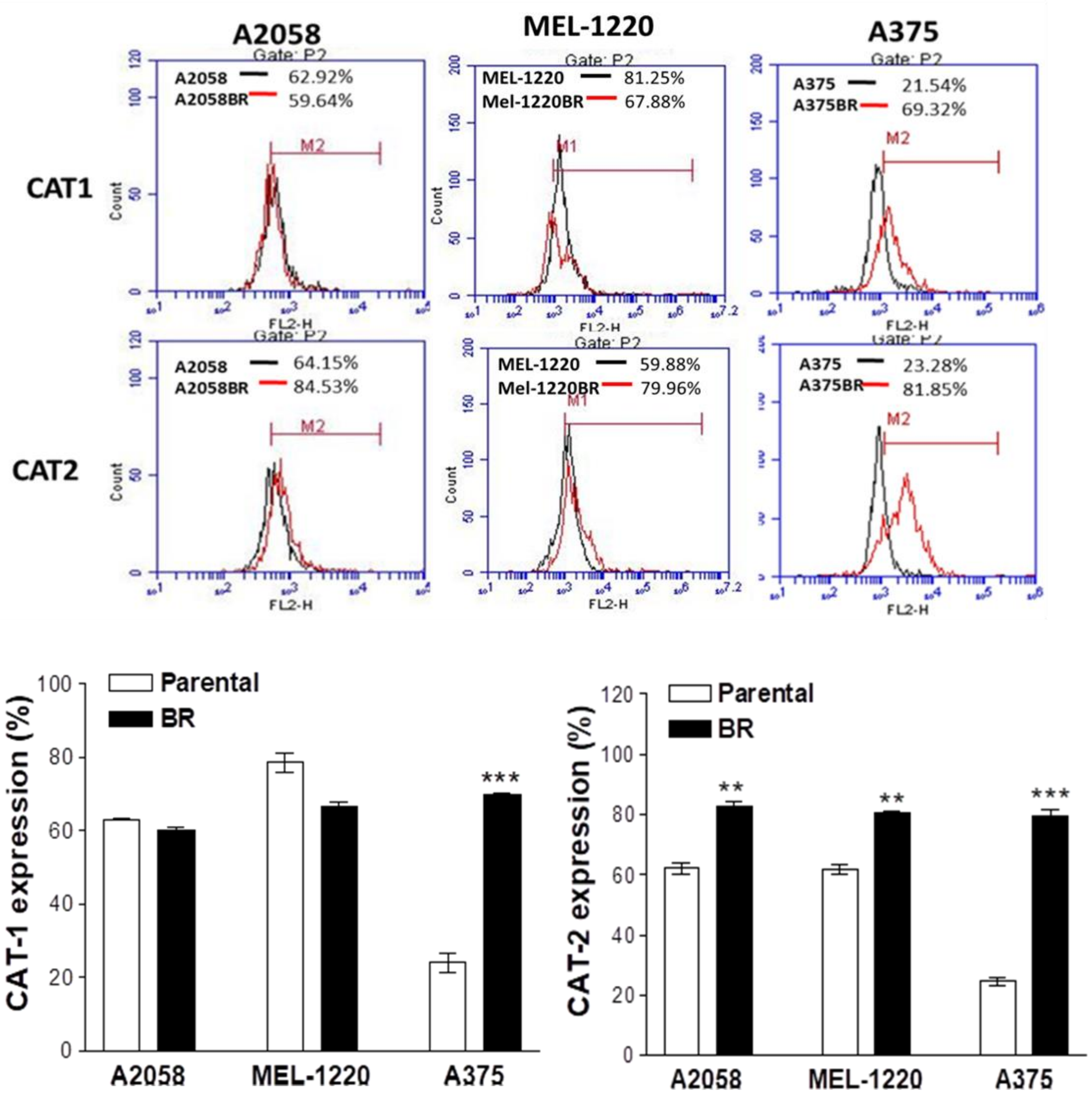

Suppl. Fig. 2. BRAFi resistant (BR) melanoma cells acquire more exogenous arginine by expressing higher levels of arginine transporter CAT-2 compared to their parental counterparts. Melanoma cells were incubated with anti-CAT-1 or anti-CAT-2 antibody and second antibody conjugated with Alexa Fluor® 555 and then analyzed by flow cytometry (FACS). The black and red curves in histograms respectively indicated parental and BR cells. The relative expression of CAT-1 or CAT-2 shown in bar graphs was based on the percentage of M1 or M2 gate. (*p < 0.05, **p < 0.01, and ***p < 0.005).

Suppl. Fig. 3

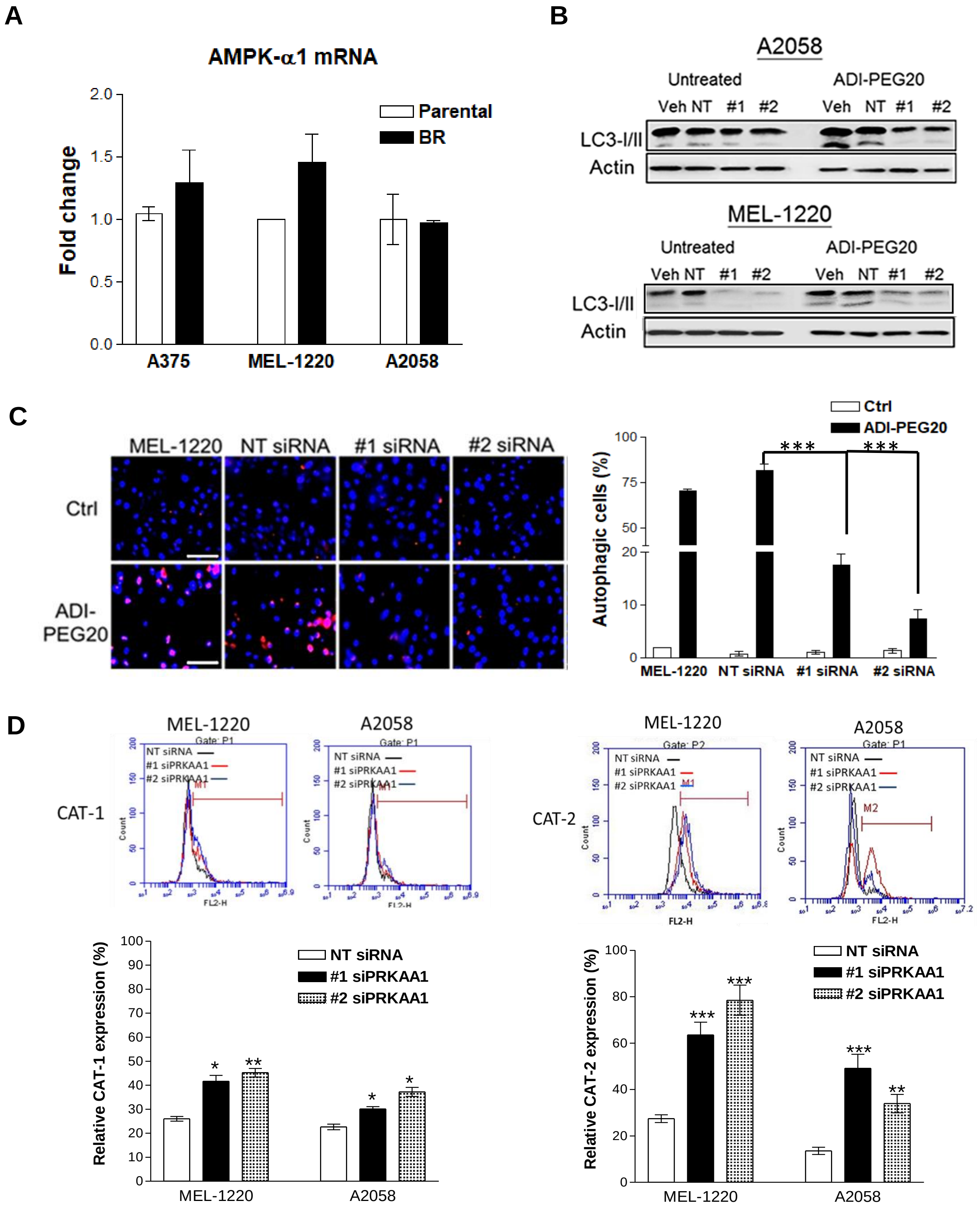

Suppl. Fig. 3. Silencing AMPK-α1 expression results in abrogation of autophagy and significant upregulation CAT-2 expression and slightly enhances CAT-1 expression in parental cells. (A) There was no significant difference of AMPK-α1 RNA levels between parental and BR cells. (B) A2058 and MEL-1220 cells were transfected with individual siRNAs against AMPK-α1 or a non-targeting (NT) siRNA (50 nM). After cultured in arginine free or completed medium (ctrl), the cell lysates of these transfectants were subjected for to immunoblot for detection of LC3-I/II expression. (C) The autophagosomes and nuclei in MEL-1220 transfectants were stained with lysotracker (red) and DAPI (blue), respectively (scale bar = 50 μm). Autophagy positive cells were quantitated and shown in a bar graph. (D) The levels of CAT-1 or CAT-2 of these transfectants were detected by anti-CAT-1 or anti-CAT-2 antibody and second antibody conjugated with Alexa Fluor® 555, and quantitated using FACS. Their relative expressions in bar graphs were based on the percentages of M1 or M2 gates in histograms (*$p < 0.05$, **$p < 0.01$, and ***$p < 0.005$).

Suppl. Fig. 4

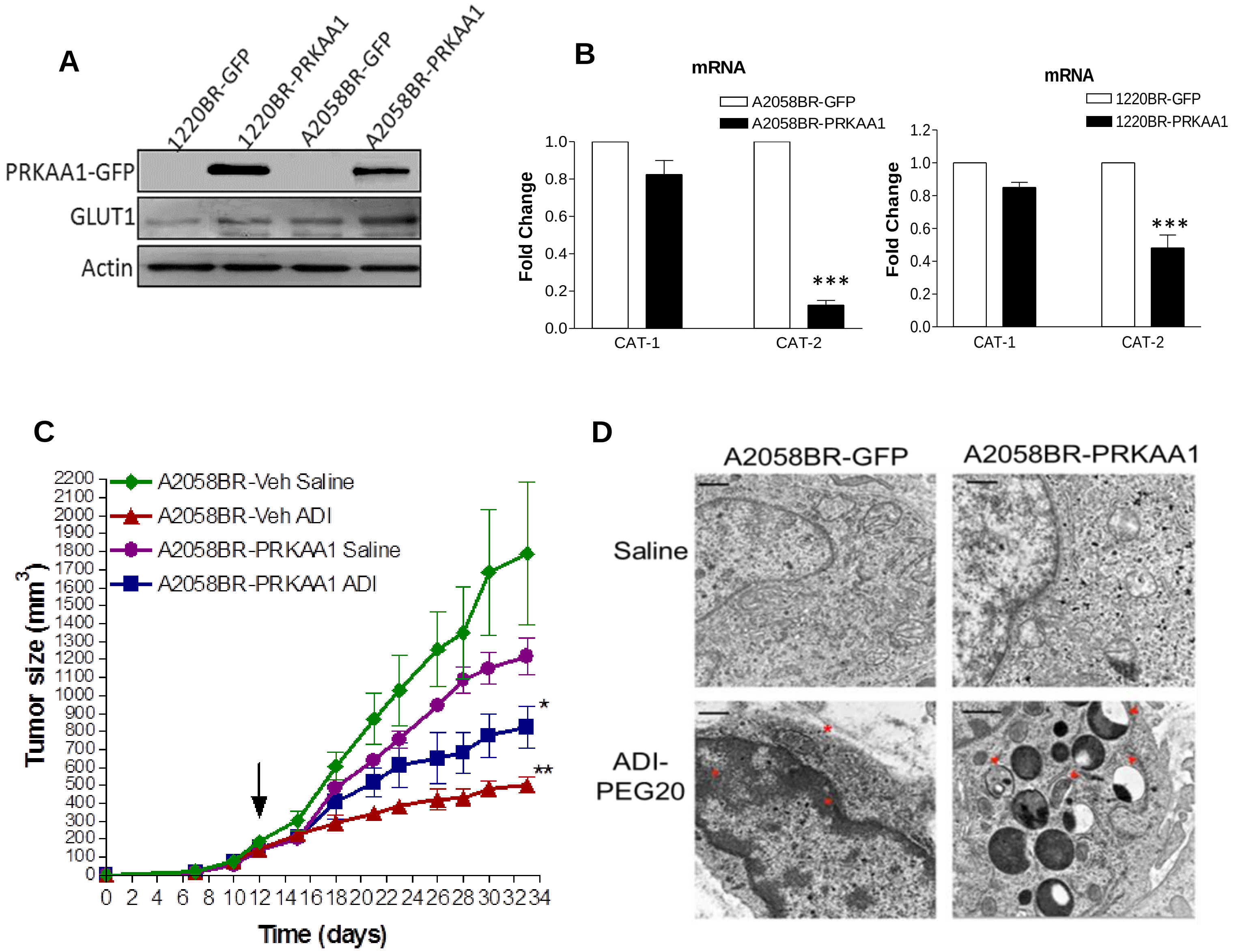

Suppl. Fig. 4. Overexpressing AMPK-α1 (PRKAA1)-GFP in BR cells restores the ability to undergo autophagy and switches acquisition of arginine to glucose. A2058BR and MEL-1220BR cells were transfected with plasmids containing AMPK-α1-GFP or GFP alone (vehicle). (A) The levels of AMPK-α1 and GLUT1 in transfectants were determined by immunoblotting. (B) CAT-1 and CAT-2 expressions were confirmed by qRT-PCR (C) 1 x 10^6 transfectants were injected into mice. Mice bearing A2058BR-GFP or A2058BR-PRKAA1 melanoma xenografts were treated with 100 ng/ml ADI-PEG20 or saline when the tumor volume reached around 100 nm^3. Tumor volumes were represented as mean $\pm$ SEM (n = 5); *p < 0.05, **p < 0.01, and ***p < 0.005. (D) *In vivo* cell death (red asterisks) and autophagy (red arrows) were visualized by TEM, 25000X. Scale bar = 500 nm.

Suppl. Fig. 5

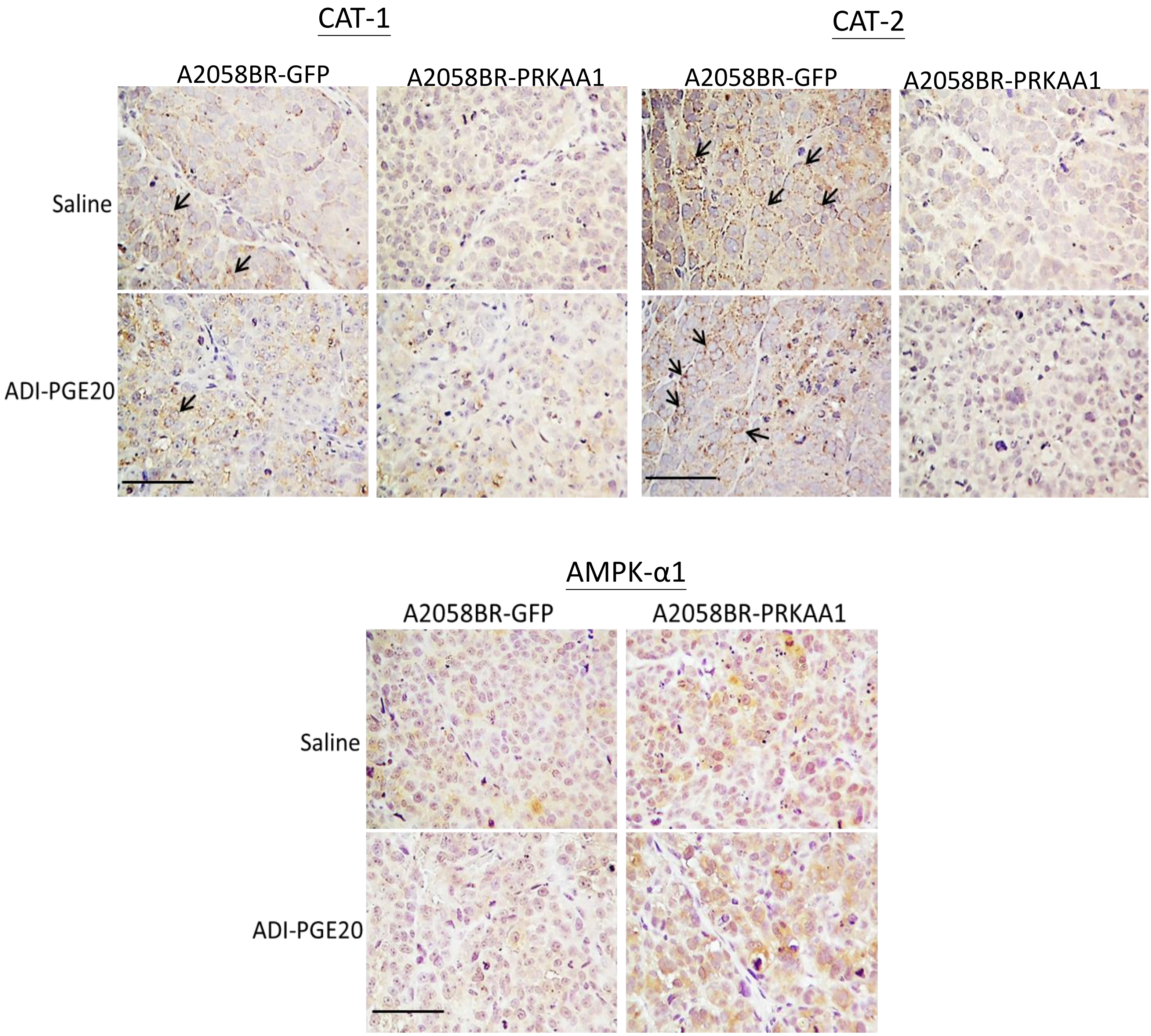

Suppl. Fig. 5. Mouse xenograft models demonstrate that overexpression of AMPK-α1 (PRKAA1) in A2058BR cells drastically reduces CAT-2 expression, corresponding to Suppl. Fig. 4. CAT-1 and CAT-2 expressions (black arrows) were detected by IHC staining (scale bar = 100 μm). A2058BR-GFP cells expressed high levels of CAT-2 and low levels of CAT-1. Decreased CAT-1 and CAT-2 expressions can be seen in A2058BR-PRKAA1 cells. AMPK-α1 staining was used to confirm PRKAA1 overexpression.

Suppl. Fig. 6

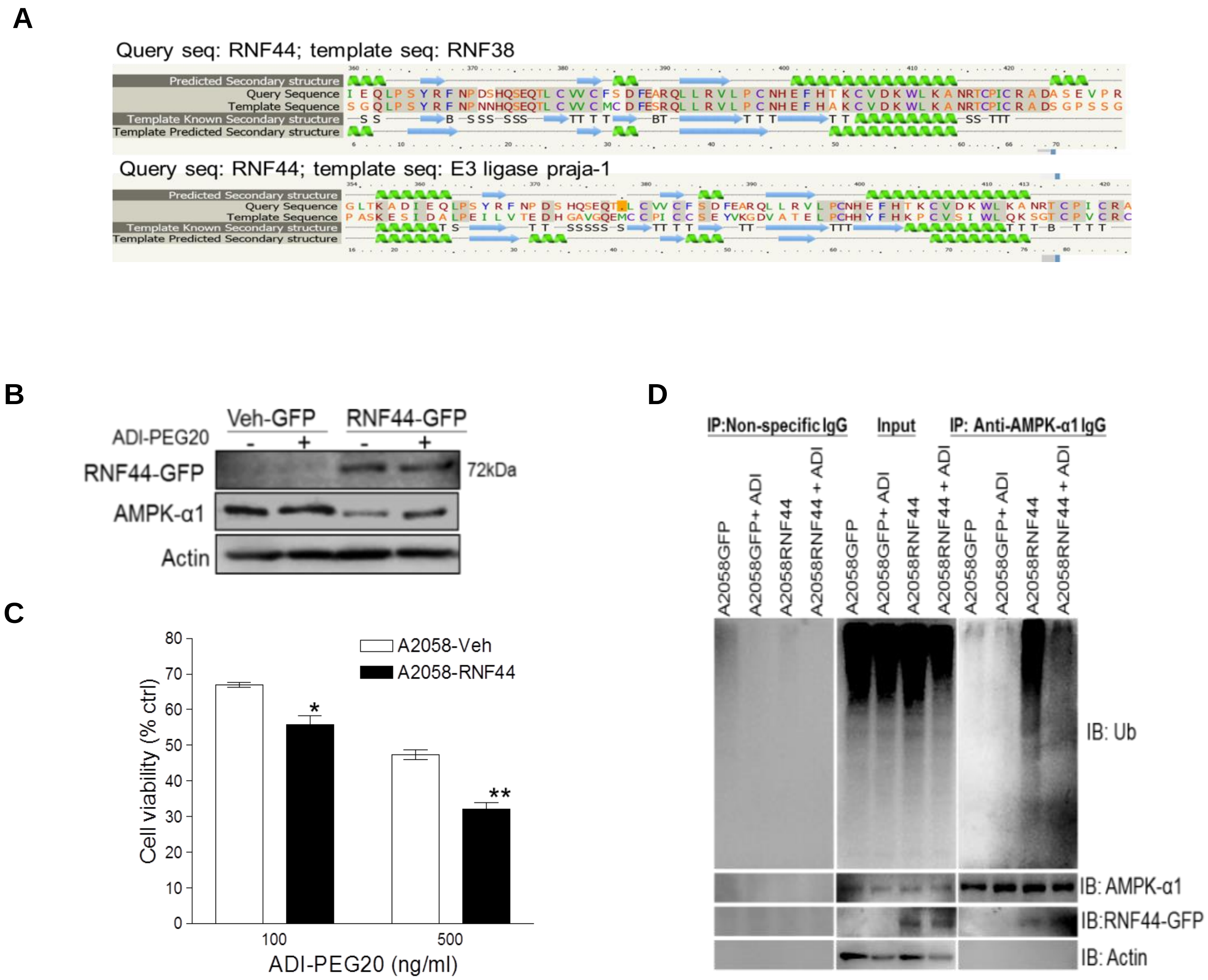

Suppl. Fig. 6. Overexpressing RNF44 in A2058 cells renders A2058 cells more sensitive to arginine deprivation due to enhanced ubiquitin-dependent AMPK-α1 degradation. (A) The partial protein sequence of RNF44 analyzed by a server Phyre2 and PDB database is similar to RNF38 and E3 ligase praja-1. (B) Overexpression of RNF44 was confirmed by immunoblotting. (C) Cell viability of A2058 transfectants which overexpress RNF44-GFP or GFP (vehicle, veh) were assayed by MTT following ADI-PEG20 (100 or 500 ng/ml) treatment for 72 hr (n = 3, *p < 0.05, **p < 0.01, and ***p < 0.005). (D) RNF44-GFP and ubiquitin (Ub) were co-immunoprecipitated with AMPK-α1, and subsequently analyzed by immunoblotting.

Suppl. Fig. 7

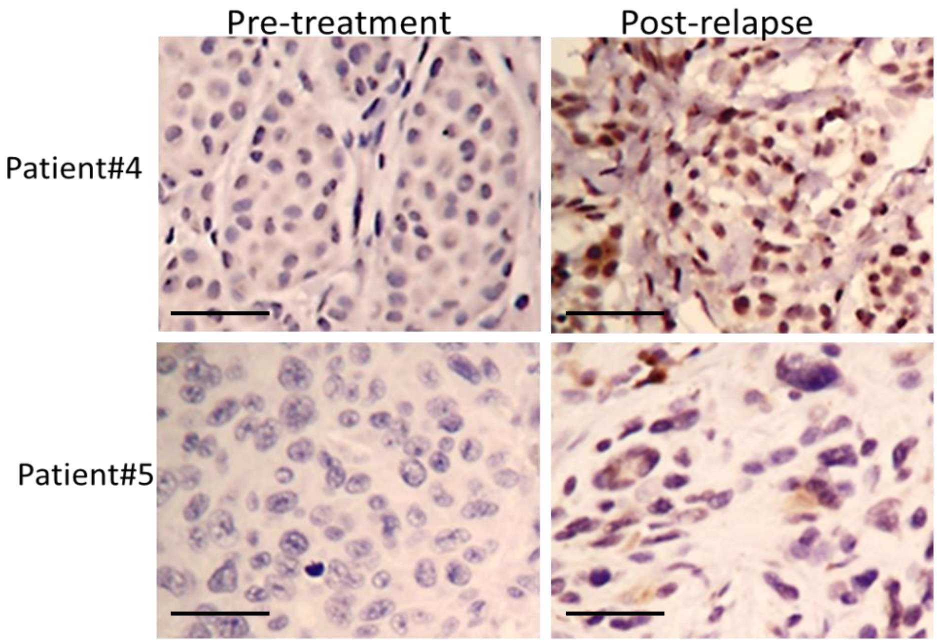

Suppl. Fig. 7. RNF44 expression appears in tumor tissues from patients who failed BRAFi (patient #4) or BRAFi/MEKi (patient #5) treatment, corresponding to Fig. 7C.RNF44 expression was detected by IHC staining (400 X) (scale bar = 50 µm).
